# High-Valent Pyrazolate-Bridged Platinum Complexes: A Joint Experimental and Theoretical Study

**DOI:** 10.1021/acs.inorgchem.2c01441

**Published:** 2022-08-03

**Authors:** Lorenzo Arnal, Daniel Escudero, Sara Fuertes, Antonio Martin, Violeta Sicilia

**Affiliations:** † Departamento de Química Inorgánica, Facultad de Ciencias, Instituto de Síntesis Química y Catálisis Homogénea (ISQCH), CSIC − Universidad de Zaragoza, Pedro Cerbuna 12, 50009 Zaragoza, Spain; ‡ Department of Chemistry, KU Leuven, Celestijnenlaan 200f − box 2404, 3001 Leuven, Belgium; § Departamento de Química Inorgánica, Escuela de Ingeniería y Arquitectura de Zaragoza, Instituto de Síntesis Química y Catálisis Homogénea (ISQCH), CSIC − Universidad de Zaragoza, Campus Rio Ebro, Edificio Torres Quevedo, 50018 Zaragoza, Spain

## Abstract

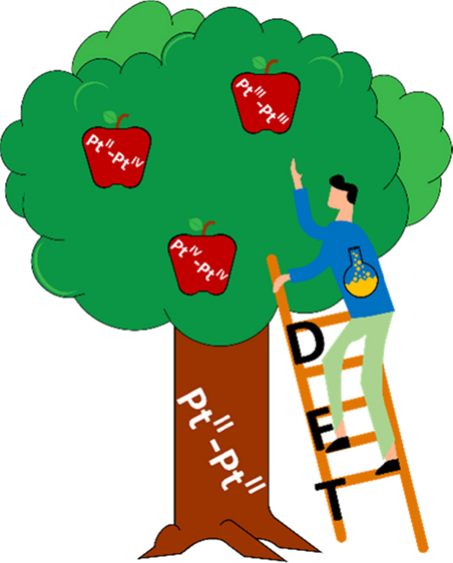

Complexes [{Pt(C^C*)(μ-pz)}_2_] (HC^C*_A_ = 1-(4-(ethoxycarbonyl)phenyl)-3-methyl-1*H*-imidazol-2-ylidene **1a**, HC^C*_B_ = 1-phenyl-3-methyl-1*H*-imidazol-2-ylidene **1b**) react with methyl iodide (MeI) at room temperature in the dark to give compounds [{Pt^IV^(C^C*)Me(μ-pz)}_2_(μ-I)]I (C^C*_A_**2a**, C^C*_B_**2b**). The reaction of **1a** with benzyl bromide (BnBr) in the same conditions afforded [Br(C^C*_A_)Pt^III^(μ-pz)_2_Pt^III^(C^C*_A_)Bn] (**5a**), which by heating in BnBr(l) became [{Pt^IV^(C^C*_A_)Bn(μ-pz)}_2_(μ-Br)]Br (**6a**). Experimental investigations and density functional theory (DFT) calculations on the mechanisms of these reactions from **1a** revealed that they follow a S_N_2 pathway in the two steps of the double oxidative addition (OA). Based on the DFT investigations, species such as [(C^C*_A_)Pt^III^(μ-pz)_2_Pt^III^(C^C*_A_)R]X (RX = MeI **Int-Me**, BnBr **Int-Bn**) and [(C^C*_A_)Pt^II^(μ-pz)_2_Pt^IV^(C^C*_A_)(R)X] (RX = MeI **Int′-Me**, BnBr **Int′-Bn**) were proposed as intermediates for the first and the second OA reactions, respectively. In order to put the mechanisms on firmer grounds, **Int-Me** was prepared as [(C^C*_A_)Pt^III^(μ-pz)_2_Pt^III^(C^C*_A_)Me]BF_4_ (**3a′**) and used to get [I(C^C*_A_)Pt^III^(μ-pz)_2_Pt^III^(C^C*_A_)Me](**4a**), [(C^C*_A_)Pt^II^(μ-pz)_2_Pt^IV^(C^C*_A_)(Me)I](**Int′-Me**), and [{Pt^IV^(C^C*)Me(μ-pz)}_2_(μ-I)]BF_4_ (**2a′**). The single-crystal X-ray structures of **2a**, **2b**, **3a′**, and **5a** along with the mono- and bi-dimensional ^1^H and ^195^Pt{^1^H} NMR spectra of all the named species allowed us to compare structural and spectroscopic data for high-valent complexes with the same core [{Pt(C^C*)(μ-pz)}_2_] but different oxidation states.

## Abstract 2

Experimental and theoretical investigations on the mechanisms of OA reactions of MeI and BnBr to [{Pt^II^(C^C*)(μ-pz)}_2_] allowed us to get high-valent pyrazolate-bridged platinum compounds: Pt_2_(III,III), Pt_2_(II,IV), and Pt_2_(IV,IV). Their stability and structural and spectroscopic features have been compared.

## Introduction

Oxidative addition (OA)–reductive elimination processes on d^8^ transition metal complexes account for many organic transformations.^1−5^ High-valent metal–metal-bonded binuclear species play very often a key role as intermediates.^6−9^ Compared to those of Rh_2_(I,I) and Ir_2_(I,I), the mechanisms of OA reactions of haloalkanes (RX) to Pt_2_(II,II) complexes have been scarcely studied. In the case of Pt_2_(II,II) complexes with the metal atoms far away from each other, the OA reactions follow monometallic pathways.^10−12^ However, if the metal centers are held in proximity by bridging ligands, different kinds of mono- or bimetallic mechanisms can operate. In lantern- or half-lantern-shaped complexes, exhibiting short intermetallic separation (<3.0 Å), it is well known that the [Pt_2_(POP)_4_]^4–^ (POP = pyrophosphite, d_Pt–Pt_ = 2.925 Å) complex undergoes thermal two-electron two-center [2e, 2c] OA of RI (R = Me, Et, ^*n*^Pr, ^*i*^Pr, n-pentyl) following a radical mechanism, although contribution of a S_N_2-type one cannot be excluded for MeI.^13^ Furthermore, the half-lantern compound [{Pt(bzq)(μ-N^S)}_2_] [bzq = benzo[*h*]quinoline, HN^S = 2-mercaptopyrimidine] also undergoes [2e, 2c] thermal OA of CH_3_I and CHX_3_ (X = Br, I) following a bimetallic S_N_2 or radical mechanism.^14^ On the other hand, complexes [Pt_2_Me_2_(C^N)_2_(μ-P^P)] [C^N = 2-phenylpyridyl-H, benzo[*h*]quinoline; P^P = dppf (1,1′-bis(diphenylphosphino)ferrocene), dppa (1,1′-bis(diphenylphosphino)acetylene)],^11,12,15^ and *cis*,*cis*-[Me_2_Pt(μ-NN)(μ-dppm)PtMe_2_] (NN = phthalazine, dppm = bis(diphenylphosphino)methane), with flexible bridging ligands, reacted with MeI in two steps, via a monometallic S_N_2 mechanism. As a result, the diplatinum(IV) derivatives [Pt_2_Me_4_I_2_(C^N)_2_(μ-P^P)] and [Me_3_Pt(μ-I)_2_(μ-dppm)PtMe_3_] were obtained.^16^

Pyrazolates are a kind of adaptative bridging ligands. Because they have a proven ability to hold two metal atoms in close proximity while enabling a wide range of intermetallic separations; they allow for different kinds of OA mechanisms. For instance, haloalkanes such as CH_3_I or CH_2_I_2_ add to [{Ir^I^(μ-pz)}_2_] complexes following mostly a bimetallic S_N_2^17−19^ or radical^20,21^ mechanisms, leading to metal–metal-bonded Ir_2_(II,II) complexes. Monometallic S_N_2^22,23^ pathways resulting in mixed valence Ir(I)–Ir(III) compounds have sometimes been proven. The Ir_2_(II,II) species, once rarely formed, undergo further OA; when this happens, no metal–metal-bonded Ir(III)–Ir(III) compounds were obtained.^24,25^ In the field of pyrazolate-bridged Pt_2_(II,II) complexes, we observed that [{Pt^II^(C^C*_A_)(μ-pz)}_2_] (HC^C*_A_ = 1-(4-(ethoxycarbonyl)phenyl)-3-methyl-1*H*-imidazol-2-ylidene **1a**) reacted with haloforms, CHX_3_ (X = Cl, Br, I), following a radical mechanism. Complexes [{Pt(C^C*_A_)(μ-pz)X}_2_], [XPt(C^C*_A_)(μ-pz)_2_Pt(C^C*_A_)CHX_2_] or mixtures of both were obtained, depending mostly on the environmental conditions (argon atmosphere, oxygen or light).^26,27^ In depth mechanistic investigations evidenced that complex **1a** exists in solution in two forms: the butterfly-wing-spreading form **1a–s** characterized by long intermetallic distances and the wing-folding one **1a–f**, with short ones. These two conformers interconvert one into the other, resembling a butterfly flapping process. Species **1a–f** are those which trigger the reaction with haloforms in the ground state (S_0_) with CHBr_3_ and CHI_3_ or in the excited state S_1_ with CHCl_3_.^27^ These results highlighted the relevance of metal–metal cooperativity to enable the oxidation of **1a**. These findings, along with the importance of high-valent organometallic complexes in many organic synthesis, encouraged us to widen the scope of our earlier investigations, exploring the reactivity of **1a** and the analogous complex [{Pt^II^(C^C*_B_)(μ-pz)}_2_] (HC^C*_B_ = 1-phenyl-3-methyl-1*H*-imidazol-2-ylidene (**1b**) toward other halogenated species, such as methyl iodide (MeI) and benzyl bromide (BnBr). Supported by density functional theory (DFT) studies on the OA mechanisms, we were able to prepare dinuclear complexes with different oxidation states: Pt_2_(III,III), Pt_2_(III,III) ↔ Pt_2_(II,IV) and Pt_2_(IV,IV). They allowed us to substantiate the modeled mechanisms and to compare their structural and spectroscopic data.

## Experimental Section

General information about instrumentation, X-ray structure determinations (CCDC 2160386–2160389), DFT computational details with Figure S1, and NMR spectra for characterization are available in the Supporting Information.

**Figure 1:**
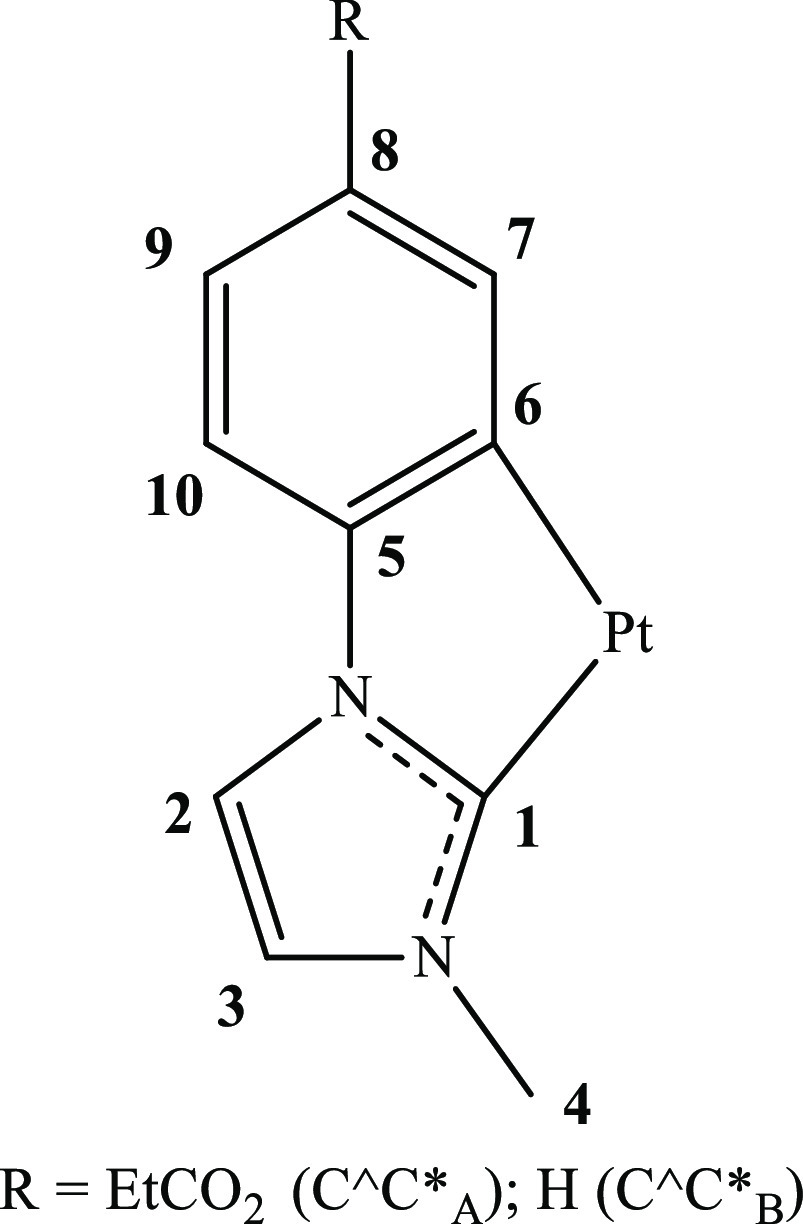
Numerical scheme for NMR purposes.

Compounds [{Pt(C^C*_A_)(μ-pz)}_2_] (**1a**),^26^ and [{Pt(C^C*_B_)(μ-Cl)}_2_] (**B**)^28^ were prepared as described elsewhere. MeI, BnBr, Hpz, and AgClO_4_ were used as purchased from Acros Organics, Fluka, Merck, and Aldrich, respectively. NMR spectra were recorded at r.t., except if a different value is indicated. Data are given according to Figure 1.

### Synthesis of [{Pt(C^C*_B_)(μ-pz)}_2_] (**1b**)

AgClO_4_ (55.1 mg, 0.263 mmol) was added to a stirred suspension of **B** (102.1 mg, 0.132 mmol) in acetone (40 mL) in the dark at room temperature. After 2.5 h, pzH (35.8 mg, 0.527 mmol) was added to the mixture and allowed to react for 18.5 h in the darkness. Then, the resulting suspension was filtered through Celite and the solution was concentrated to 50 mL. Afterward, NEt_3_ (0.5 mL, 3.62 mmol) was added to the solution at r.t. and allowed to react for 2 h. The suspension was concentrated to 15 mL, and the solid was filtered and washed with 2 mL of acetone to give **1b** as a white solid. Yield: 74.0 mg, 0.088 mmol, 67%. Anal. calcd for C_26_H_24_N_8_Pt_2_: C, 37.23; H, 2.88; N, 13.36. Found: C, 37.22; H, 2.98; N, 12.97. ^1^H NMR (400 MHz, DMSO-d_6_): δ = 7.43 (d, ^3^*J*_2,3_ = 1.6, 2H, H_2_), 7.25 (s, 2H, H_pz_), 7.16 (s, 2H, H_pz_), 6.82 (d, *J*_H,H_ = 7.4, 2H, H_Ar_), 6.76 (d, 2H, H_3_), 6.63 (d, *J*_H,H_ = 7.3, 2H, H_Ar_), 6.50 (t, *J*_H,H_ = 7.4, 2H, H_Ar_), 6.35 (t, *J*_H,H_ = 7.0, 2H, H_Ar_), 5.88 (s br, 2H, H_pz_), 2.72 (s, 6H, H_4_). ^1^H–^195^Pt HMQC NMR (85.6 MHz, DMSO-d_6_): δ = −3767.4 (s).

### Synthesis of [{Pt(C^C*_A_)Me(μ-pz)}_2_(μ-I)]I (**2a**)

CH_3_I (26 μL, 0.403 mmol) was added to a solution of **1a** (98.9 mg, 0.101 mmol) in anhydrous CH_2_Cl_2_ (5 mL) under argon atmosphere in the dark. After 14 h of reaction, the precipitate was filtered, washed with Et_2_O (4 × 10 mL), and dried to give **2a** as a white solid. Yield: 117.8 mg; 0.093 mmol; 92%. Anal. calcd for C_34_H_38_I_2_N_8_O_4_Pt_2_: C, 32.24; H, 3.02; N, 8.85. Found: C, 31.84; H, 2.82; N, 8.45. ^1^H NMR (400 MHz, CD_2_Cl_2_, 248 K): δ = 7.97 (s, ^3^*J*_H,Pt_ = 37.7, 2H, H_7_), 7.94 (d, ^3^*J*_9,10_ = 8.2, 2H, H_9_), 7.87–7.73 (m, 6H, H_pz_ and H_2_), 7.47 (d, ^3^*J*_10, 9_ = 8.2, 2H, H_10_), 7.34 (d, ^3^*J*_3,2_ = 1.8, 2H, H_3_), 6.50 (s br, 2H, H_pz_), 4.27 (q, ^3^*J*_H,H_ = 6.9, 4H, OC*H_2_*CH_3_), 3.52 (s, 6H, H_4_), 1.78 (s, ^2^*J*_H,Pt_ = 65.5, 6H, Pt-C*H*_3_), 1.31 (t, ^3^*J*_H,H_ = 6.9, 6H, OCH_2_C*H_3_*). ^13^C{^1^H} NMR plus HMBC and HSQC (101 MHz, CD_2_Cl_2_, 248 K): δ = 165.7 (s, 2C, C=O), 146.3 (s, 2C, C_5_), 142.1 (s, 2C, ^1^*J*_C,Pt_ = 1126.2, C_1_), 140.1 and 139.2 (4C, C_pz_), 133.3 (s, 2C, C_7_), 128.4 (s, 2C, C_9_), 125.5 (s, 1C, C_3_), 117.5 (s, 1C, C_2_), 113.6 (s, 2C, C_10_), 107.6 (s, 2C, C_pz_), 61.5 (s, 2C, O*C*H_2_CH_3_), 38.1 (s, 2C, C_4_), 14.3 (s, 2C, OCH_2_*C*H_3_), 11.6 (s, ^1^*J*_C,Pt_ = 502.2, 2C, Pt-*C*H_3_). ^195^Pt{^1^H} NMR (85.6 MHz, CD_2_Cl_2_, 248 K): δ = −2688.0 (s). MS (MALDI+): *m*/*z* = 1138.88 [{Pt(C^C*_A_)(CH_3_)(μ-pz)}_2_(μ-I)]^+^. IR (ATR, cm^–1^) υ = 1698 (m, C=O).

### Synthesis of [{Pt(C^C*_B_)Me(μ-pz)}_2_(μ-I)]I (**2b**)

CH_3_I (18 μL, 0.2862 mmol) was added to a suspension of **1b** (60 mg, 0.072 mmol) in anhydrous DMF (5 mL) under argon atmosphere in the dark. After 8 h of reaction, 100 mL of Et_2_O was added and the precipitate was filtered, washed with Et_2_O (5 × 10 mL), and dried to give **2b** as a white solid. Yield: 70.7 mg; 0.063 mmol; 88%. Anal. calcd for C_28_H_30_I_2_N_8_Pt_2_: C, 29.96; H, 2.69; N, 9.98. Found: C, 29.76; H, 2.62; N, 9.65. ^1^H NMR (400 MHz, CD_2_Cl_2_): δ = 7.78 (m, 4H, H_pz_), 7.70 (d, ^3^*J*_2,3_ = 2.0, 2H, H_2_), 7.41–7.24 (m, 8H, H_3_ and H_Ar_), 7.13 (m, 2H, H_Ar_), 6.47 (m, 2H, H_pz_), 3.52 (s, 6H, H_4_), 1.80 (s, ^2^*J*_H,Pt_ = 66.1, 6H, Pt-C*H*_3_). ^1^H–^195^Pt HMQC NMR (85.6 MHz, CD_2_Cl_2_): δ = −2664.4 (s). MS (MALDI+): *m*/*z* = 994.07 [{Pt(C^C*_B_)(CH_3_) (μ-pz)}_2_(μ-I)]^+^.

### Synthesis of [(C^C*_A_)Pt(μ-pz)_2_Pt(C^C*_A_)Me]BF_4_ (**3a′**)

Me_3_OBF_4_ (49.3 mg, 0.320 mmol) was added to a solution of **1a** (262.0 mg, 0.267 mmol) in anhydrous CH_2_Cl_2_ (15 mL) under argon atmosphere in the dark at −25 °C. After 2 h of reaction, the solution was dried in vacuo. The residue was treated with 20 mL of dried Et_2_O, and the resulting solid was filtered, washed with Et_2_O (2 × 20 mL), and dried to give **3a′** as a brown solid. Yield: 244.8 mg; 0.226 mmol; 85%. Anal. calcd for C_33_H_35_BF_4_N_8_O_4_Pt_2_·2CH_2_Cl_2_: C, 33.51; H, 3.13; N, 8.93. Found: C, 33.27; H, 3.10; N, 9.33. ^1^H NMR (400 MHz, CD_2_Cl_2_): δ = 7.92 (d, ^3^*J*_H,H_ = 2.1, 1H, H_pz_), 7.85–7.77 (m, 3H, 2H_pz_, and H_9_ [Pt–Me]), 7.74 (d, ^3^*J*_H,H_ = 2.1, 1H, H_pz_), 7.66 (dd, ^3^*J*_9,10_ = 8.2, ^4^*J*_9,7_ = 1.6, 1H, H_9_ [Pt]), 7.52 (d, ^4^*J*_7,9_ = 1.6, ^3^*J*_H,Pt_ = 41.2, 1H, H_7_[Pt]), 7.50 (d, ^4^*J*_7,9_ = 1.6, ^3^*J*_H,Pt_ = 51.7, 1H, H_7_ [Pt–Me]), 7.43 (d, ^3^*J*_2,3_ = 2.0, 1H, H_2_ [Pt–Me]), 7.24–7.16 (m, 2H, H_10_ [Pt–Me] and H_2_ [Pt]), 7.03 (d, ^3^*J*_10,9_ = 8.1, 1H, H_10_ [Pt]), 6.60–6.52 (m, 3H, 2H_pz_ and H_3_), 6.37 (d, ^3^*J*_3,2_ = 2.1, 1H, H_3_ [Pt]), 4.38–4.23 (m, 4H, OC*H_2_*CH_3_), 3.17 (s, 3H, H_4_ [Pt–Me]), 3.05 (s, 3H, H_4_ [Pt]), 2.42 (s, ^2^*J*_H,Pt_ = 70.7, ^3^*J*_H,Pt_ = 14.7, 3H, [Pt-C*H*_3_]), 1.40–1.30 (m, 6H, OCH_2_C*H_3_*). ^13^C{^1^H} NMR plus HMBC and HSQC (101 MHz, CD_2_Cl_2_): δ = 166.5 (s, 1C, C=O), 166.0 (s, 1C, C=O), 153.8 (s, 1C, ^1^*J*_C,Pt_ = 1391.4, C_1_ [Pt]), 150.4 (s, 1C, C_5_ [Pt]), 147.2 (s, 1C, C_5_ [Pt–Me]), 144.5 (s, 1C, ^1^*J*_C,Pt_ = 1179.2, C_1_ [Pt–Me]), 140.2 (s, 1C, C_pz_), 137.9 (s, 1C, C_pz_), 135.9 and 135.5 (s, 4C, C_pz_), 134.7 (s, 1C, C_7_ [Pt]), 133.2 (s, 1C, C_7_ [Pt–Me]), 129.8 (s, 1C, C_9_ [Pt–Me]), 129.6 (s, 1C, C_9_ [Pt]), 124.8 (s, 1C, C_3_ [Pt–Me]), 123.6 (s, 1C, C_3_ [Pt]), 117.4 (s, 1C, C_2_ [Pt–Me]), 116.8 (s, 1C, C_2_ [Pt]), 113.7 (s, 1C, C_10_ [Pt–Me]), 112.1 (s, 1C, C_10_ [Pt]), 107.8 and 107.7 (s, 2C, C_pz_), 61.9 and 61.8 (s, 2C, O*C*H_2_CH_3_), 37.2 (s, 1C, C_4_ [Pt–Me]), 37.0 (s, 1C, C_4_ [Pt]), 14.6 and 14.5 (s, 2C, OCH_2_*C*H_3_), −1.9 (s, ^1^*J*_C,Pt_ = 411.2, 1C, Pt-*C*H_3_).^19^F NMR (376 MHz, CD_2_Cl_2_): δ = −151.4 (m, 4F, BF_4_). ^195^Pt{^1^H} NMR (85.6 MHz, CD_2_Cl_2_): δ = −2589.2 (s, ^1^*J*_Pt,Pt_ = 1023.4, Pt–Me), −3064.2 (s, Pt). MS (MALDI+): *m*/*z* = 997.2 [(C^C*_A_)Pt(μ-pz)_2_Pt(C^C*_A_)CH_3_]^+^. IR (ATR, cm^–1^) υ = 1702 (m, C=O), 1043, 1012 and 519 (s, BF_4_).

### Synthesis of [I(C^C*_A_)Pt(μ-pz)_2_Pt(C^C*_A_)Me] (**4a**)

KI (33.6 mg, 0.202 mmol) was added to a solution of **3a′** (109.8 mg, 0.101 mmol) in MeCN (3 mL) in the dark at −25 °C. After 3 h of reaction, the suspension was filtered and the solid was washed with water (7 × 10 mL) and dried to give **4a** as a yellow solid. Yield: 44.0 mg; 0.039 mmol; 39%. Anal. calcd for C_33_H_35_IN_8_O_4_Pt_2_: C, 35.54; H, 3.14; N, 9.96. Found: C, 35.88; H, 2.88; N, 9.65. ^1^H NMR (400 MHz, CD_2_Cl_2_, 223 K): δ = 8.01 (d, ^3^*J*_H,H_ = 2.0, 1H, H_pz_), 7.89 (d, ^3^*J*_H,H_ = 2.0, 1H, H_pz_), 7.72 (d, ^3^*J*_H,H_ = 2.0, 1H, H_pz_), 7.64 (d, ^3^*J*_H,H_ = 2.0, 1H, H_pz_), 7.61 (dd, ^3^*J*_9,10_ = 8.2, ^4^*J*_9,7_ = 1.6, 1H, H_9_ [Pt–Me]), 7.53 (d, ^4^*J*_7,9_ = 1.6, ^3^*J*_H,Pt_ = 58.7, 1H, H_7_ [Pt–I]), 7.44–7.33 (m, 2H, H_9_ [Pt–I] and H_7_[Pt–Me]), 7.04 (d, ^3^*J*_2,3_ = 2.1, 1H, H_2_ [Pt–Me]), 6.89 (d, ^3^*J*_10,9_ = 8.2, 1H, H_10_ [Pt–Me]), 6.84 (d, ^3^*J*_2,3_ = 2.1, 1H, H_2_ [Pt–I]), 6.93 (d, ^3^*J*_10,9_ = 8.2, 1H, H_10_ [Pt–I]), 6.38–6.30 (m, 3H, H_pz_ and H_3_ [Pt–Me]), 6.15 (d, ^3^*J*_3,2_ = 2.0, 1H, H_3_ [Pt–I]), 4.35–4.10 (m, 4H, OC*H_2_*CH_3_), 3.04 (s, 3H, H_4_ [Pt–I]), 3.01 (s, 3H, H_4_ [Pt–Me]), 1.48 (s, ^2^*J*_H,Pt_ = 61.0, ^3^*J*_H,Pt_ = 14.5, 3H, Pt-C*H*_3_), 1.32–1.24 (m, 6H, OCH_2_C*H_3_*). ^13^C{^1^H} NMR plus HMBC and HSQC (101 MHz, CD_2_Cl_2_, 223 K): δ = 166.3 (s, 1C, C=O), 165.8 (s, 1C, C=O), 153.0 (s, ^1^*J*_C,Pt_ = 1358.7, 1C, C_1_ [Pt–I]), 147.8 (s, 1C, C_5_), 147.5 (s, ^1^*J*_C,Pt_ = 1153.6, 1C, C_1_ [Pt–Me]), 145.7 (s, 1C, C_5_), 140.5, 139.1 and 134.3 (s, 3C, C_pz_), 132.9 (s, 1C, C_7_ [Pt–I]), 132.0 (s, 1C, C_7_ [Pt–Me]), 131.7 (s, 1C, C_pz_), 128.1, 127.1, 126.2 and 125.2 (C_6_, C_6_, C_8_ and C_8_), 126.4 (s, 1C, C_9_ [Pt–Me]), 125.3(s, 1C, C_9_ [Pt–I]), 123.0 (s, 1C, C_3_ [Pt–Me]), 121.7 (s, 1C, C_3_ [Pt–I]), 114.1 (s, 1C, C_2_ [Pt–Me]), 113.7 (s, 1C, C_2_ [Pt–I]), 111.1 (s, 1C, C_10_ [Pt–Me]), 110.0 (s, 1C, C_10_ [Pt–I]), 105.6 (m, 2C, C_pz_), 61.1 and 60.9 (s, 2C, O*C*H_2_CH_3_), 36.6 and 36.5 (s, 2C, C_4_, [Pt–Me] and [Pt–I]), 14.1 (s br, 2C, OCH_2_*C*H_3_), −16.0 (s, ^1^*J*_C,Pt_ = 467.1, 1C, Pt-*C*H_3_). ^195^Pt{^1^H} NMR (85.6 MHz, CD_2_Cl_2_, 223 K): δ = −2848.2 (s, ^1^*J*_Pt,Pt_ = 1239.8, Pt–Me), −3018.8 (s, Pt–I). MS (MALDI+): *m*/*z* = 997.2 [(C^C*_A_)Pt(μ-pz)_2_Pt(C^C*_A_)(CH_3_)] ^+^. IR (ATR, cm^–1^) υ = 1699 (m, C=O).

### Synthesis of **Int′-Me**

PPh_4_I (83.4 mg, 0.179 mmol) was added to a solution of **3a′** (97.0 mg, 0.089 mmol) in anisole (5 mL) in the dark at 30 °C, and the mixture was allowed to react for 2 h. Then, the solvent was removed under vacuum and the residue was treated with H_2_O. The resulting yellow solid was identified as [(C^C*_A_)Pt(μ-pz)_2_(C^C*_A_)PtI(CH_3_)] **(Int′-Me)** by ^1^H and ^195^Pt NMR, although anisole and PPh_4_BF_4_ were detected as impurities. ^1^H NMR (400 MHz, 223 K, CD_2_Cl_2_): δ = 8.10–6.00 (16 H_Ar_ of **Int′-Me** and H_Ar_ of PPh_4_), 4.33–4.07 (m, 4H, OC*H_2_*CH_3_), 3.75 (s, OC*H*_3_, anisole), 3.49 and 3.19 (s, 6H, H_4_), 1.75 (s, ^2^*J*_H,Pt_ = 65.9, 3H, [Pt-C*H*_3_]). ^195^Pt{^1^H} NMR (85.6 MHz, 223 K, CD_2_Cl_2_): δ = −2697.0 (s, Pt^IV^), −3776.0 (s, Pt^II^).

### Synthesis of [Br(C^C*_A_)Pt(μ-pz)_2_Pt(C^C*_A_)Bn] (**5a**)

BnBr (33 μL, 0.277 mmol) was added to a solution of **1a** (68.0 mg, 0.069 mmol) in MeCN (20 mL) in the dark. After 3.5 h of reaction, the solvent was removed under vacuum. The residue was treated with a mixture of Et_2_O and *n*-hexane (1:20) to give **5a** as an orange solid. Yield: 74.0 mg; 0.064 mmol; 93%. Anal. calcd for C_39_H_39_BrN_8_O_4_Pt_2_: C, 40.60; H, 3.41; N, 9.71. Found: C, 40.32; H, 3.36; N, 9.68. ^1^H NMR (400 MHz, CD_2_Cl_2_, 248 K): δ = 7.97 (d, ^3^*J*_H,H_ = 1.8, 1H, H_pz_), 7.91 (d, ^3^*J*_H,H_ = 1.8, 1H, H_pz_), 7.78 (d, ^3^*J*_H,H_ = 2.1, 1H, H_pz_), 7.65 (dd, ^3^*J*_9,10_ = 8.2, ^4^*J*_9,7_ = 1.7, 1H, H_9_ [Pt–Bn]), 7.50 (d, ^4^*J*_7,9_ = 1.7, ^3^*J*_H,Pt_ = 43.3, 1H, H_7_ [Pt–Bn]), 7.44 (d, ^4^*J*_7,9_ = 1.7, ^3^*J*_H,Pt_ = 45.2, 1H, H_7_ [Pt–Br]), 7.39 (dd, ^3^*J*_9,10_ = 8.2, ^4^*J*_9,7_ = 1.7, 1H, H_9_ [Pt–Br]), 7.16 (d, ^3^*J*_H,H_ = 2.1, 1H, H_pz_), 7.06–6.98 (m, 1H, H_*para*_), 6.89–6.80 (m, 5H, H_2_, H_2_, H_10_ [Pt–Bn] and H_*meta*_), 6.78–6.68 (m, 3H, H_10_ [Pt–Br] and H_*orto*_), 6.40 (pt, ^3^*J*_H,H_ = 2.0, 1H, H_pz_), 6.30 (pt, ^3^*J*_H,H_ = 2.1, 1H, H_pz_), 6.11 (d, ^3^*J*_3,2_ = 2.1, 1H, H_3_ [Pt–Bn]), 6.07 (d, ^3^*J*_3,2_ = 2.1, 1H, H_3_ [Pt–Br]), 4.36–4.10 (m, 5H, CH_2_ (Bn) and OC*H_2_*CH_3_), 3.73 (d, ^2^*J*_H,H_ = 7.9, ^2^*J*_H,Pt_ = 69.0, ^3^*J*_H,Pt_ = 28.9, 1H CH_2_ (Bn)), 3.07 (s, 3H, H_4_ [Pt–Br]), 2.71 (s, 3H, H_4_ [Pt–Bn]), 1.32 (t, ^3^*J*_H,H_ = 7.3, 3H, OCH_2_C*H_3_*), 1.25 (t, ^3^*J*_H,H_ = 7.3, 3H, OCH_2_C*H_3_*).^13^C{^1^H} NMR plus HMBC and HSQC (101 MHz, CD_2_Cl_2_, 248 K): δ = 166.5 (s, 1C, C=O), 166.0 (s, 1C, C=O), 153.2 (s, ^1^*J*_C,Pt_ = 1323.4, 1C, C_1_ [Pt–Br]), 148.7 (s, ^1^*J*_C,Pt_ = 1239.5, 1C, C_1_ [Pt–Bn]), 148.1 (s, 1C, C_5_ [Pt–Br]), 147.1 (s, 1C, C_*ipso*_), 145.8 (s, 1C, C_5_ [Pt–Bn]), 138.9, 137.2 and 134.4 (s, 3C, C_pz_), 133.3 (s, 1C, C_7_ [Pt–Br]), 132.1 (s, 1C, C_7_ [Pt–Bn]), 131.9 (s, 1C, C_pz_), 128.9 (s, 2C, C_*meta*_), 127.0 and 126.9 (2C, C_9_ [Pt–Bn] and C_*orto*_), 125.7 (s, 1C, C_9_ [Pt–Br]), 124.5 (s, 1C, C_*para*_), 122.7 (s, 1C, C_3_ [Pt–Bn]), 121.9 (s, 1C, C_3_ [Pt–Br]), 114.1 and 113.9 (2C, C_2_), 111.4 (s, 1C, C_10_ [Pt–Bn]), 110.1 (s, 1C, C_10_ [Pt–Br]), 105.8 and 105.7 (2C, C_pz_), 61.3 and 61.0 ( 2C, O*C*H_2_CH_3_), 36.6 (s, 1C, C_4_ [Pt–Br]), 36.1 (s, 1C, C_4_ [Pt–Bn]), 15.2 (s, ^1^*J*_C,Pt_ = 448.1, ^2^*J*_C,Pt_ = 192.9, 1C, Pt-*C*H_2_Ph), 14.3 (s, 2C, OCH_2_*C*H_3_). ^195^Pt{^1^H} NMR (85.6 MHz, CD_2_Cl_2_, 248 K): δ = −2693.6 (s br, Pt–Bn), −2742.8 (s, ^1^*J*_Pt,Pt_ = 1028.9, Pt–Br,). MS (MALDI+): *m*/*z* = 1061.6 [Br(EtO_2_C-C^C*)Pt(μ-pz)_2_Pt(EtO_2_C-C^C*)]^+^, 1072.8 [(C^C*_A_)Pt(μ-pz)_2_Pt(C^C*_A_)Bn]^+^. IR (ATR, cm^–1^) υ = 1700 (m, C=O).

### Synthesis of [{Pt(C^C*_A_)Bn(μ-pz)}_2_(μ-Br)]Br (**6a**)

A suspension of **5a** (95 mg, 0.082 mmol) in BnBr (5 mL) was heated 70 °C for 5 h. Then, the suspension was cooled down to room temperature, and the resulting solid was filtered and dried to give **6a**. Yield: 94.7 mg; 0.071 mmol; 87%. Anal. calcd for C_46_H_46_Br_2_N_8_O_4_Pt_2_**:** C, 41.70; H, 3.50; N, 8.46. Found: C, 41.38; H, 3.27; N, 8.44. ^1^H NMR (400 MHz, CD_2_Cl_2_, 248 K): δ = 8.46 (s br, 2H, H_pz_), 8.35 (s br, 2H, H_pz_), 8.10 (d, ^4^*J*_7,9_ = 1.1, ^3^*J*_H,Pt_ = 37.5, 2H, H_7_), 7.89 (dd, ^3^*J*_9,10_ = 8.2, ^4^*J*_9,7_ = 1.1, 2H, H_9_), 7.18 (s, 2H, H_2_), 7.15–7.03 (m, 6H, H_10_, H_*para*_ and H_3_), 6.88 (pt, *J*_H,H_ = 7.6, 4H, H_*meta*_), 6.79 (s br, 2H, H_pz_), 6.64 (pd *J*_H,H_ = 7.6, 4H, H_*orto*_), 4.45–4.20 (m, 6H, CH_2_ (Bn) and OC*H_2_*CH_3_), 4.02 (d, ^2^*J*_H,H_ = 8.6, ^2^*J*_H,Pt_ = 90.6, 2H, CH_2_ (Bn)), 3.41 (s, 6H, H_4_), 1.35 (t, ^3^*J*_H,H_ = 7.3, 6H, OCH_2_C*H_3_*). ^13^C{^1^H} NMR plus HMBC and HSQC (101 MHz, CD_2_Cl_2_, 248 K): δ = 165.7 (s, 2C, C=O), 146.4 (s, 2C, C_5_), 142.6 (s, 2C, ^1^*J*_C,Pt_ = 1202.2, C_1_), 141.1 (2C, C_pz_), 141.0 (s, 2C, C_*ipso*_), 138.4 (2C, C_pz_), 132.2 (s, 2C, C_7_), 128.8 (s, 2C, C_9_), 128.7 (s, 2C, C_*meta*_), 128.3 (s, 4C, ^3^*J*_C,Pt_ = 23.0, C_*orto*_), 127.1 (s, 2C, C_*para*_), 124.5 (s, 2C, C_3_), 116.5 (s, 2C, C_2_), 113.6 (s, ^4^*J*_C,Pt_ = 28.7, 2C, C_10_), 107.4 (s, ^3^*J*_C,Pt_ = 18.0, 2C, C_*pz*_), 61.5 (s, 2C, O*C*H_2_CH_3_), 37.6 (s, 2C, C_4_), 32.7 (s, ^2^*J*_C,Pt_ = 521.4, 2C, Pt-*C*H_2_Ph), 14.3 (s, 2C, OCH_2_*C*H_3_). ^195^Pt{^1^H} NMR (85.6 MHz, CD_2_Cl_2_, 248 K): δ = −2357.0 (s). MS (MALDI+): *m*/*z* = 1245.69 [{Pt(C^C*_A_)Bn(μ-pz)}_2_(μ-Br)]^+^. IR (ATR, cm^–1^) υ = 1715 (m, C=O).

## Results and Discussion

### Reactivity of [{Pt^II^(C^C*)(μ-pz)}_2_](C^C*_A_**1a**, C^C*_B_**1b**) with MeI and BnBr: Experimental and Computational Investigations for the Mechanistic Studies

**Scheme 1:**
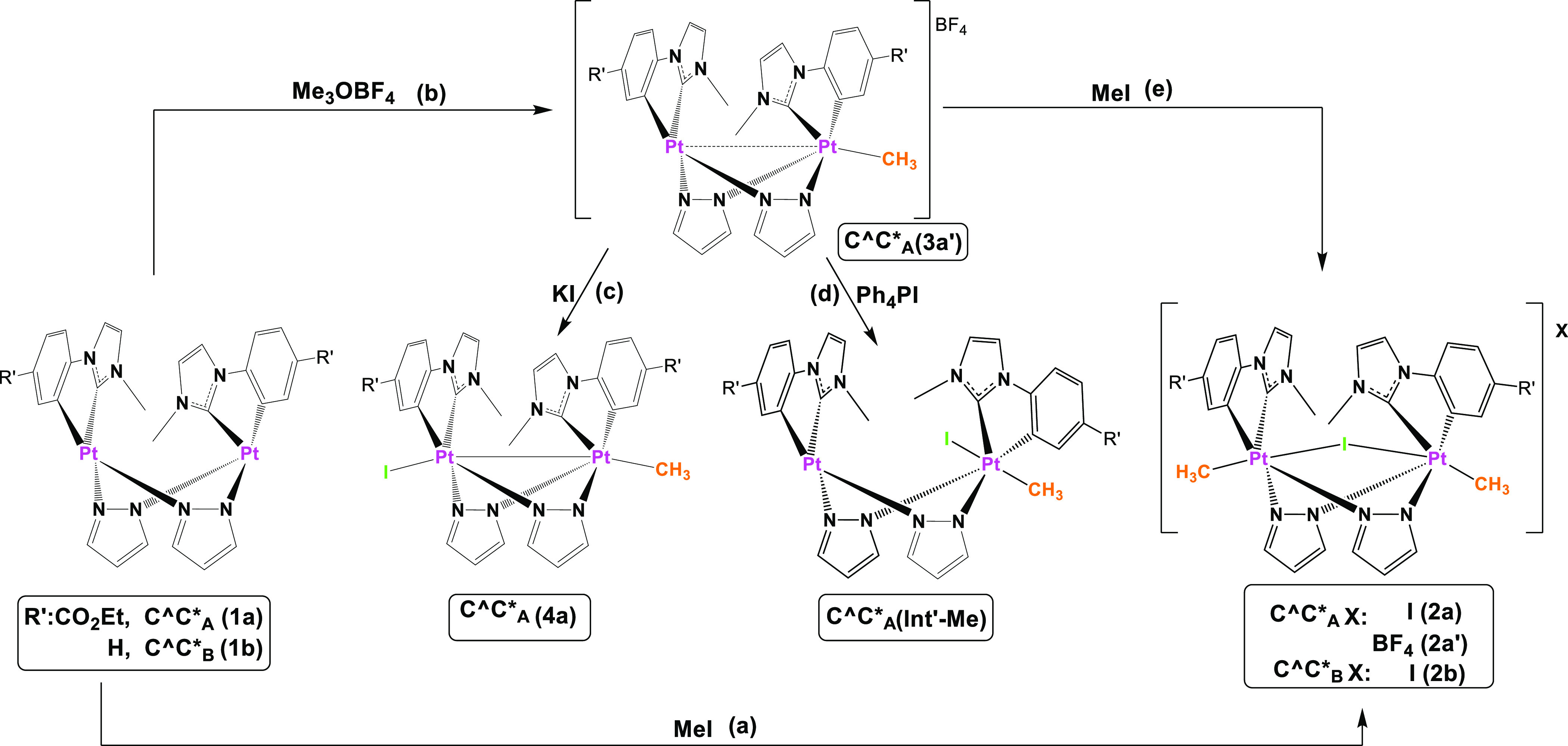
Reaction Pathway for OA Reactions of MeI to **1a** and **1b**

The reaction of [{Pt^II^(C^C*_A_)(μ-pz)}_2_] (**1a**) with MeI in MeCN in the dark afforded the Pt_2_(IV,IV) compound [{Pt^IV^(C^C*_A_)Me(μ-pz)}_2_(μ-I)]I (**2a**) as result of a double OA of MeI, regardless the reactant molar ratio (Scheme 1, path a).

The use of other solvents such as acetone or dichloromethane does not change the nature of the final compound. Compound [I(C^C*_A_)Pt^III^(μ-pz)_2_Pt^III^(C^C*_A_)Me] (**4a**), resulting from the bimetallic OA of one MeI molecule was just detected by ^1^H NMR. To evaluate if the CO_2_Et fragment plays a role in the redox behavior of complex **1a**, we prepared the new complex **1b** (Experimental Section in the Supporting Information and Figure S2). Then, it was reacted with MeI in DMF due to its low solubility in other organic solvents, giving rise to the Pt_2_(IV,IV) complex [{Pt^IV^(C^C*_B_)Me(μ-pz)}_2_(μ-I)]I (**2b**). This result indicates that the CO_2_Et fragment does not affect the reactivity of these Pt_2_(II,II) complexes toward MeI; however, it increases their solubility, allowing for a better study of it. Complexes **2a** and **2b** were isolated as white solids in very good yields (92%, **2a**; 88%, **2b**) and fully characterized (Figures S3 and S4). Just two complexes with the same bridging system have been reported to date, PPN[{Pt^IV^Me_3_(μ-pz)}_2_(μ-I)]^29^ and (PPh_4_)[{Pt^IV^Me_2_Br(μ-pz)}_2_(μ-Br)],^30^ both of them prepared by assembly of mononuclear Pt^IV^ fragments. Therefore, **2a** and **2b** are the first Pt_2_(IV,IV) derivatives obtained by OA of MeI to {Pt^II^(μ-pz)}_2_ fragments.

**Scheme 2:**
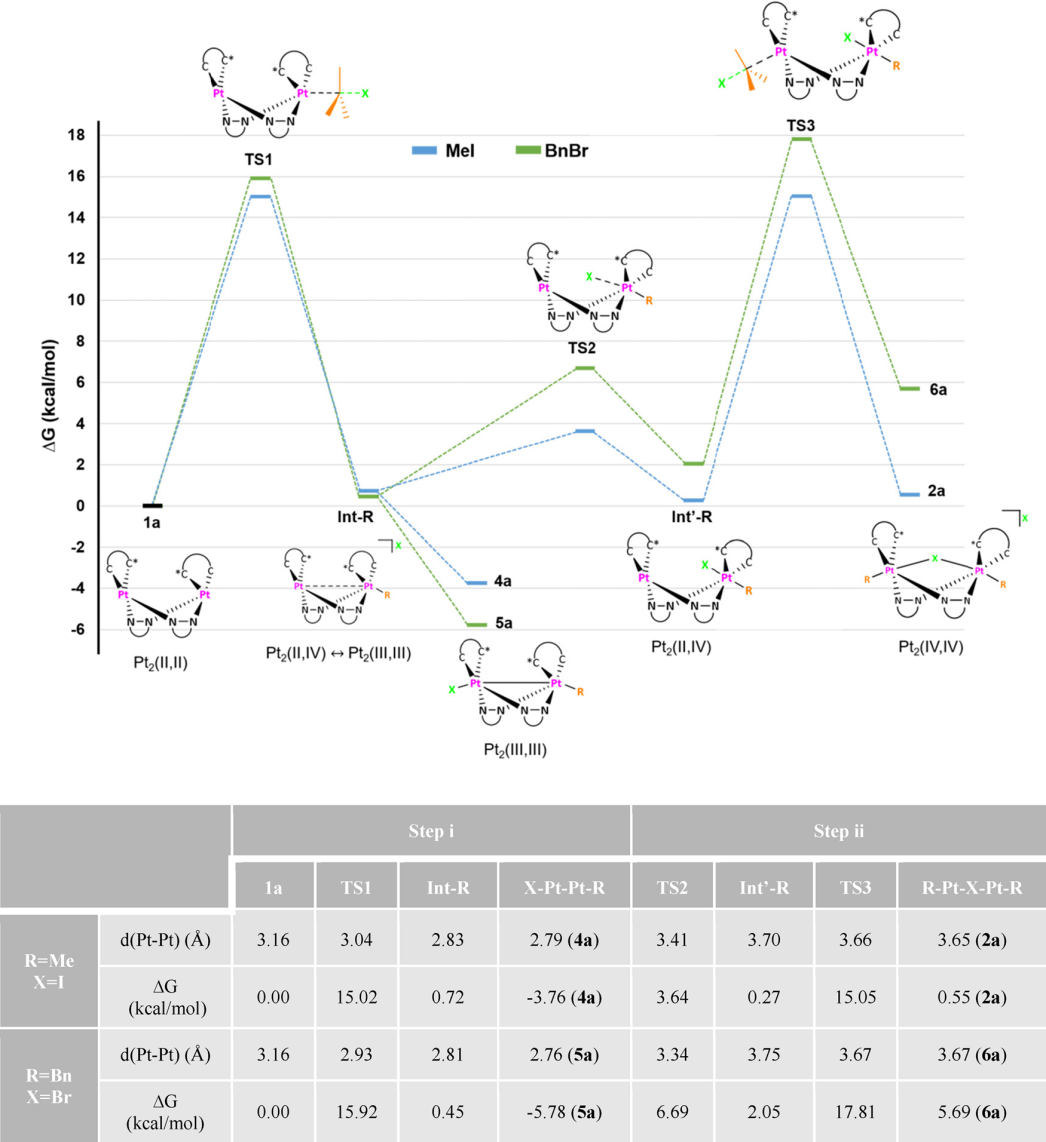
Computed (PCM(MeCN)-M06/6-31G(d), MWB60(Pt), and MWB46(I)) Free Energy Profile (Δ*G*, kcal/mol) for the Thermal Conversion of **1a** into **4a** (or **5a**) and **Int′-R** (Step i) and **Int′-R** into **2a** (or **6a**) (Step ii) Following S_N_2 Mechanisms

Keeping in mind that compound **1a** is oxidized by CHX_3_ (X = Br, I) in the dark through a radical mechanism,^26,27^ we checked this possibility for MeI. The reaction of **1a** with MeI in MeCN-d_3_ in the dark was performed with and without galvinoxyl (Gal·) as radical (R·) trap, and they were followed by ^1^H NMR for 1 h. It resulted to be almost unaffected by the presence of Gal·(see Figure S5), which led us to dismiss a radical mechanism and to consider a S_N_2 one for the first and the second OA of MeI to **1a**. For an in depth knowledge of these reaction mechanisms, we carried out a DFT study (see Computational Details in Supporting Information). The free energy profiles in MeCN have been represented in Scheme 2, the reference energy value being 0.0 kcal/mol for one of the Pt_2_(II,II) reactant, **1a**.

In the modeled mechanism, the first OA is a S_N_2 reaction, Nu + MeI → NuMe^+^ + I^–^, with the dinuclear compound **1a** acting as nucleophile (Nu) to give a cationic [Pt(II)–Pt(IV) −Me]^+^ intermediate **Int-Me**. The reaction would proceed through a transition state TS1, with one imaginary frequency (436i cm^–1^), which shows a hypervalent C atom with two long Pt···C and C···I distances. The energy barrier (Ea_TS1_ = 15.02 kcal/mol) is low enough to allow the reaction to proceed at room temperature in the dark. Once the intermediate **Int-Me** was formed, the migration of the halide to the Pt(II) center will afford the I–Pt(III) −Pt(III) −Me derivative (**4a**) while, if the halide bonds to the Pt(IV) center, Pt(II,IV) species (**Int′-Me**) will be generated.

The small barrier (2.92 kcal/mol) for the conversion of **Int-Me** into **Int′-Me** through the transition state TS2 (43i cm^–1^) competes with the barrierless formation of **4a** (Figure S6). This along with the low free energy difference between **4a** and **Int′-Me** (Δ*G*_Int′-Me-4a_ = 4.03 kcal/mol) support the formation of the two species, **4a** and **Int′-Me** from **Int-Me**, which are believed to be in equilibrium in solution of MeCN at r.t.

The second OA reaction (Scheme 2) would start with the nucleophilic attack of the d_z_^2^ orbital of the Pt(II) center in complex **Int′-Me** to a second MeI molecule to give **2a** as the final product. This step could proceed through a transition state TS3 (423i cm^–1^), with the energy barrier (Ea_TS3_ = 14.78 kcal/mol) being similar to that of the first OA and thus small enough to be surpassed at room temperature in the dark. Therefore, this calculated mechanism shows the feasible access to **Int′-Me**, which would explain the observed double OA of MeI to **1a** to give **2a**. Besides, it shows that **4a** is thermodynamically more stable than **2a**. Because of this, the scarce solubility of the latter in the reaction media is likely to be the driving force for **2a**, which will be the final product of the reaction of **1a** with MeI.

Species like **Int-Me** and **Int′-Me** have been proposed as intermediates in OA reactions of one or two RX molecules to M_2_(I,I) (M = Rh, Ir).^17,25^ Besides, the mixed-valence species **Int′-Me** could also be available by a monometallic S_N_2 OA of MeI to **1a**.^31^ Aiming to test the proposed mechanism and to compare the structural and spectroscopic features of high-valent Pt_2_ complexes, with the same core “{Pt(C^C*_A_)(μ-pz)}_2_” but with different oxidation states, we addressed the synthesis and characterization of additional compounds such as **3a′**, **4a**, and **Int′-Me** (Scheme S1 and Figures S7–S10).

**Scheme 3:**
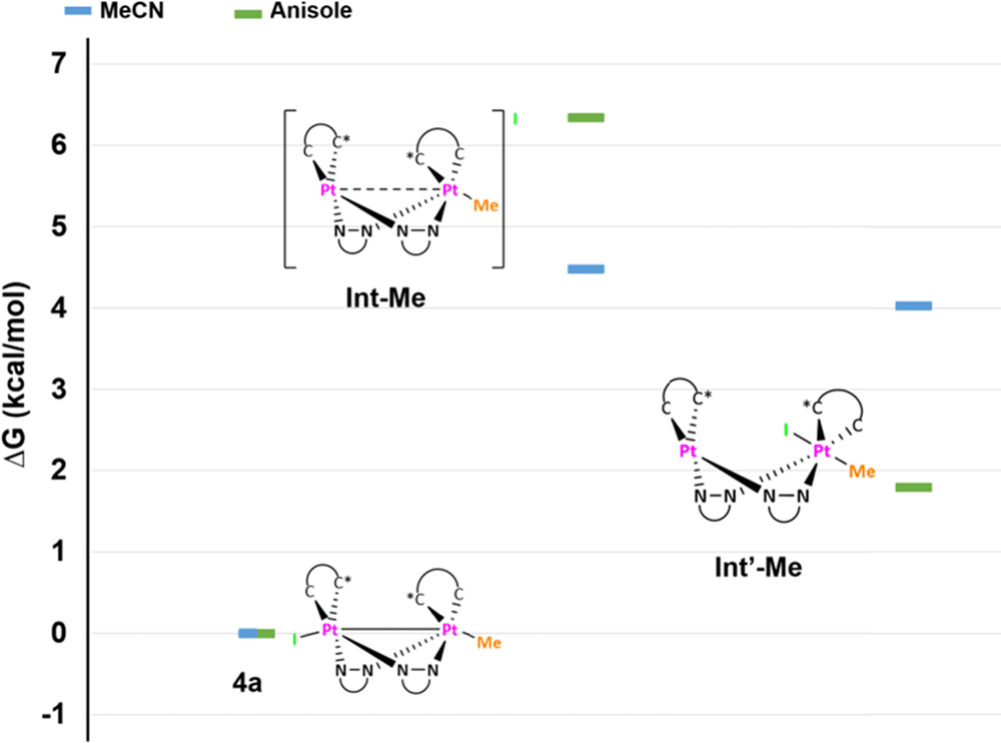
Computed (PCM-M06/6-31G(d), MWB60(Pt), and MWB46(I)) Free Energy Profiles (Δ*G*, kcal/mol) in MeCN (ε = 35.688) and Anisole (ε = 4.2247) for the Thermal Conversion of **4a**, **Int-Me**, and **Int′-Me**

First, to achieve our challenging tasks, **Int-Me** was prepared as the BF_4_ salt, [(C^C*_A_)Pt(μ-pz)_2_Pt(C^C*_A_)Me]BF_4_ (**3a′**), in a very good yield (85%) (see Scheme 1, path b) by reacting **1a** with Me_3_OBF_4_, at −25 °C in anhydrous CH_2_Cl_2_ in the dark, under argon atmosphere. Compound **3a′** resulted to be stable in the solid state and solution at room temperature and could be fully characterized (Figure S7). Then, **3a′** was reacted with KI in MeCN at low temperature (−25 °C) to favor the exothermic process (Scheme 1, path c). In these conditions, [I(C^C*_A_)Pt^III^(μ-pz)_2_Pt^III^(C^C*_A_)Me] (**4a**) precipitated in the reaction media and could be obtained as a pure species in a moderate yield (39%) and then characterized (Figure S8). A mixture of **4a** and **Int′-Me** remains in the mother liquor, as it was detected by ^1^H NMR, which explains the low yield in the synthetic procedure. Further support for the simultaneous formation of both **4a** and **Int′-Me** along with the equilibrium between them was obtained following this reaction by NMR, as can be seen in Figure S9. At −30 °C, this reactions leads to the simultaneous formation of **4a** and **Int′-Me**, with the former being the major species, which becomes **Int′-Me** as the temperature raises, in such a way that after 24 h at room temperature, both species are present in the mixture in about a 1:1 molar ratio. To reach **Int′-Me** as pure species, we searched for solvents that give a smaller free energy difference between **Int′-Me** and **4a** than the one obtained in MeCN, so as to ensure a larger amount of **Int′-Me** in the equilibrium. As can be seen in Scheme 3, the computed Δ*G*_Int′-Me-4a_ in anisole (1.79 kcal/mol) is clearly smaller than that in MeCN (4.03 kcal/mol). Accordingly, [(C^C*_A_)Pt^II^(μ-pz)_2_Pt^IV^(C^C*_A_)(Me)I] (**Int′-Me**) was the single organometallic species detected by ^1^H NMR in the reaction of **3a′** with Ph_4_PI in anisole in the dark at 30 °C (Scheme 1, path d), although it was obtained from the reaction mixture unpurified with anisole and Ph_4_PBF_4_ (see Experimental section in the Supporting Information and Figure S10).

Additionally, **3a′** was reacted with MeI in acetonitrile at r.t., rendering **2a′** as the final product (Scheme 1, path e). This result is consistent with Pt_2_(III,III) ↔ Pt_2_(II,IV) formulations for **3a′**, the contribution of the Pt_2_(II,IV) one being significant. Therefore, since all the intermediate species in the double OA of MeI to **1a** resulted to be experimentally available, the proposed mechanism, initiated with a bimetallic OA of MeI to the Pt_2_(II,II) complexes **1a** and **1b**, seems suitable.

**Scheme 4:**
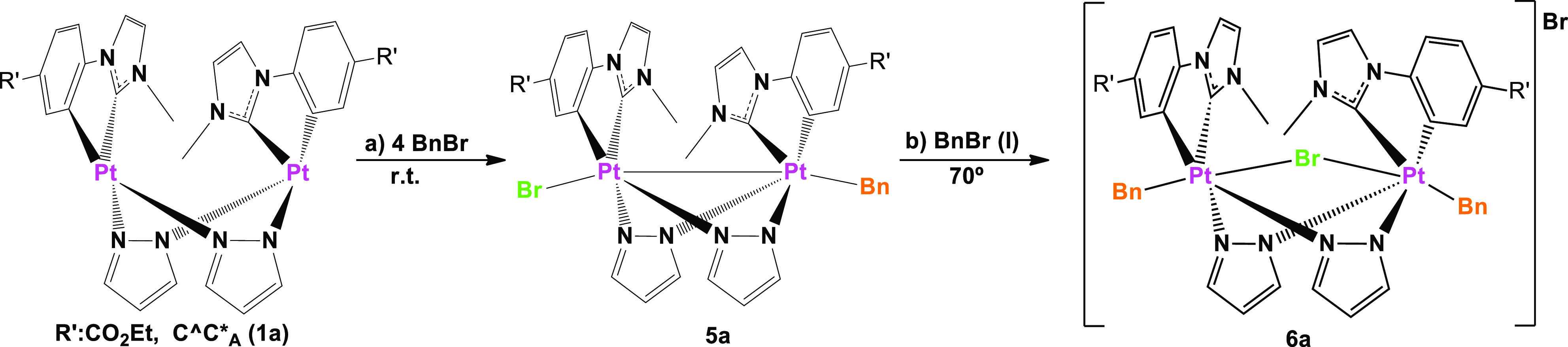
Reaction Pathway for OA Reactions of BnBr to **1a**

To expand these studies, we focused on the OA of benzyl bromide (BnBr) to [{Pt^II^(C^C*_A_)(μ-pz)}_2_] (**1a**). The reaction of **1a** with BnBr in a 1:4 molar ratio in MeCN in the dark at r.t. rendered the Pt_2_(III,III) complex [Br(C^C*_A_)Pt^III^(μ-pz)_2_Pt^III^(C^C*_A_)Bn] (**5a**) (Scheme 4, path a), which was isolated as an orange solid in very good yield (93%).

A second OA to give compound [{Pt^IV^(C^C*)Bn(μ-pz)}_2_(μ-Br)]Br (**6a**) was achieved by heating **5a** at 70 °C in BnBr(l) in the dark for 5 h. In these hard conditions, **6a** was obtained in good yield (87%) (Scheme 4, path b). Then, **5a** and **6a** were fully characterized (Figures S11 and S12). The selective formation of **5a** in the presence of oxygen, an efficient radical trap, points to a S_N_2 mechanism, like in the case of MeI (Figure S13), which was modeled by DFT in MeCN. For comparison, the free energy profiles obtained are represented in Scheme 2 and Figure S6.

The energy barrier for the first OA (Ea_TS1_ = 15.92 kcal/mol, TS1: 294i cm^–1^) is low enough to enable the reaction go at r.t. in the dark, which is not much different from that for MeI. Once the **Int-Bn** was formed, species **5a** or **Int′-Bn** becomes available. Thermodynamically, the formation of **5a** from **1a** is clearly favored (calculated Δ*G*_5a–1a_ = −5.78 kcal/mol; Δ*G*_Int′-Bn-1a_ = 2.05 kcal/mol). Although the energy barrier (6.24 kcal/mol) for conversion of **Int-Bn** into **Int′-Bn** through TS2 (44i cm^–1^) is in principle not large enough to prevent it to occur, experimentally, **5a** is the only species formed at r.t. in the dark. Therefore, it seems that the free energy difference between **5a** and **Int′-Bn** (calculated Δ*G*_Int′Bn-5a_ = 7.83 kcal/mol) hinders significant formation of **Int′-Bn**, thus preventing the second OA to occur at r.t. Only by heating at 70 °C in BnBr(l) is the formation of **Int′-Bn** achieved, enabling it to convert into **6a** through TS3 (260i cm^–1^).

Again, in view of the lower stability of **6a** compared to **5a**, the scarce solubility of the former in the reaction media is likely the driving force for its formation.

The characterization of all these Pt_2_ compounds has been addressed together for an overall perspective, as can be seen below.

### Characterization of All New High-Valent {Pt(μ-pz)}_2_ Complexes

**Figure 2:**
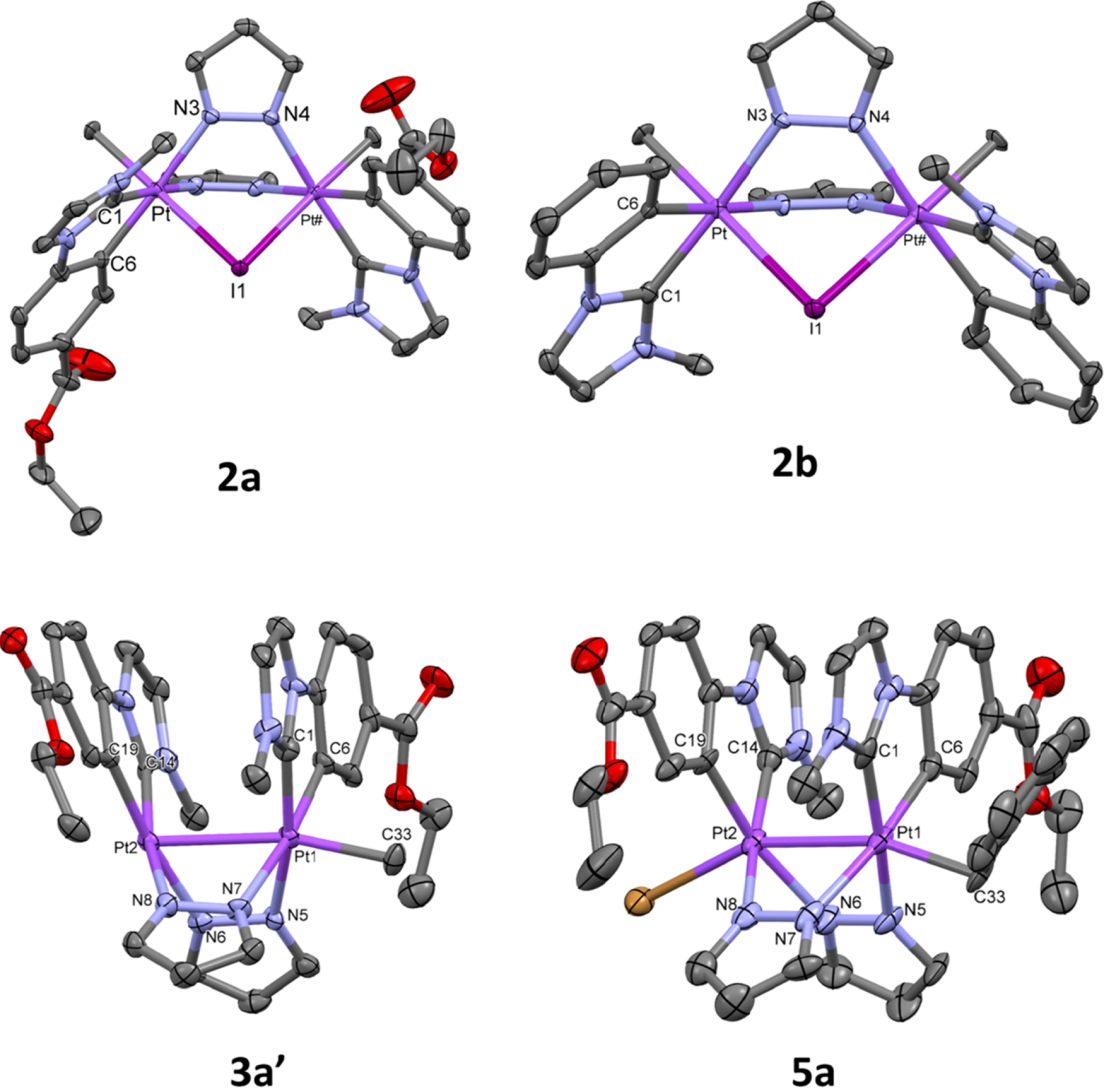
Molecular structure of the cationic complexes **2a**, **2b**, **3a′**, and **5a**. Ellipsoids are drawn at their 50% probability level; solvent molecules, I^–^ (**2a**, **2b**), BF_4_^–^ (**3a′**), and hydrogen atoms have been omitted for clarity.

In addition to elemental analysis, the most valuable information for the full characterization of these new complexes came from their ^1^H and ^195^Pt{^1^H} NMR spectra in solution (Figures S2–S4 and S7–S12). All these Pt_2_ complexes, except **1a/b** and **3a′**, are not stable in solution at r.t. without excess of RX in the media. Thus, the characterization of all these has been carried out at low temperature. Besides, single-crystal X-ray diffraction studies on complexes **2a**, **2b**, **3a′**, and **5a** have been carried out. Their molecular structures are depicted in Figure 2, and selected bond distances and angles are given in Tables S2 and S3.

As it can be seen in Figure 2, in all of them, the Pt_2_N_4_ rings exhibit a boat-like shape (angle between the Pt–N–N–Pt fragments being 73.0° **2a**, 71.7° **2b**, 89.32° **3a′**, and 89.64° **5a**) with an anti-configuration of the C^C* groups (C1–Pt–Pt#–C#1 torsion angles: 96.7(4)° **2a**, 96.1(2)° **2b**; C1–Pt1–Pt2–C14 torsion angles: 79.29° **3a′**, 72.92° **5a**).

The molecular structures of the cationic complexes, [{Pt^IV^(C^C*)Me(μ-pz)}_2_(μ-I)]^+^, in **2a** and **2b** consist of a Pt_2_(IV,IV) core bridged by two pyrazolates and one iodide ligand. The intermetallic distances (d_Pt–Pt_: 3.5909(9) Å **2a**, 3.6228(6) Å **2b**) are in between the observed ones in the Pt_2_(IV,IV) compounds (PPN)[{Pt^IV^Me_3_(μ-pz)}_2_(μ-I)] (d_Pt–Pt_: 3.706(1) Å)^29^ and (PPh_4_) [{Pt^IV^Me_2_Br(μ-pz)}_2_(μ-Br)] (d_Pt–Pt_: 3.593(1) Å).^30^ The Pt^IV^ centers exhibit octahedral PtIN_2_C_3_ coordination environments with the axial positions occupied by one Me group and the iodine bridge, the Pt–I–Pt angle being close to 80° (81.55(2) **2a**, 82.69(13) **2b**). The Pt–I, Pt–N, and Pt–C distances seem to be not affected by the metal oxidation state since they are quite similar to those observed in Pt_2_(III,III) complexes containing the same kind of ligands.^26,27^

The cationic complex [Pt_2_(C^C*_A_)_2_(μ-pz)_2_Me]^+^ in **3a′** and **5a** show short intermetallic distances (d_Pt–Pt_: 2.6700(4) Å **3a′**, 2.6545(5) Å **5a**) indicative of the existence of a Pt–Pt bond in each of them. All bond distances and angles are very similar to those observed in analogous complexes with the [(C^C*)Pt^III^(μ-pz)]_2_ core and octahedral environment at each Pt center.^26,27^ In **3a′**, the platinum centers show different coordination environments: octahedral for Pt1 with the Pt2 and the methyl group (C33) in the *apex* positions and distorted square pyramidal for Pt2, with Pt1 in the *apex* position. The intermetallic distance in **3a′** is in the range reported for “Pt_2_^III^(μ-L)_2_R” species, no matter if they exhibit an octahedral environment of each Pt center [2.529(1)–2.7910(2) Å]^32,33^ or octahedral geometry at one and square pyramidal at the other center. The latter is exemplified by complex [R(H_3_N)_2_Pt(μ-L-*N*,*O*)_2_Pt(NH_3_)_2_]^3+^ (L-*N*,*O*- = amidate or pyridonate) [2.676(1)–2.7542(11) Å].^34−41^

Regarding pyrazolate-bridged complexes, the intermetallic distance in **3a′** is a little longer than those in [(CHX_2_)(C^C*)Pt^III^(μ-pz)Pt^III^(C^C*)X],^26,27^ (d_Pt–Pt_ = 2.6302(4) Å X = Br; 2.6324(3) Å X = I) or in **5a**, which can be attributed to the larger *trans* influence of CH_3_ compared to CHX_2_ and CH_2_Ph.

**Figure 3:**
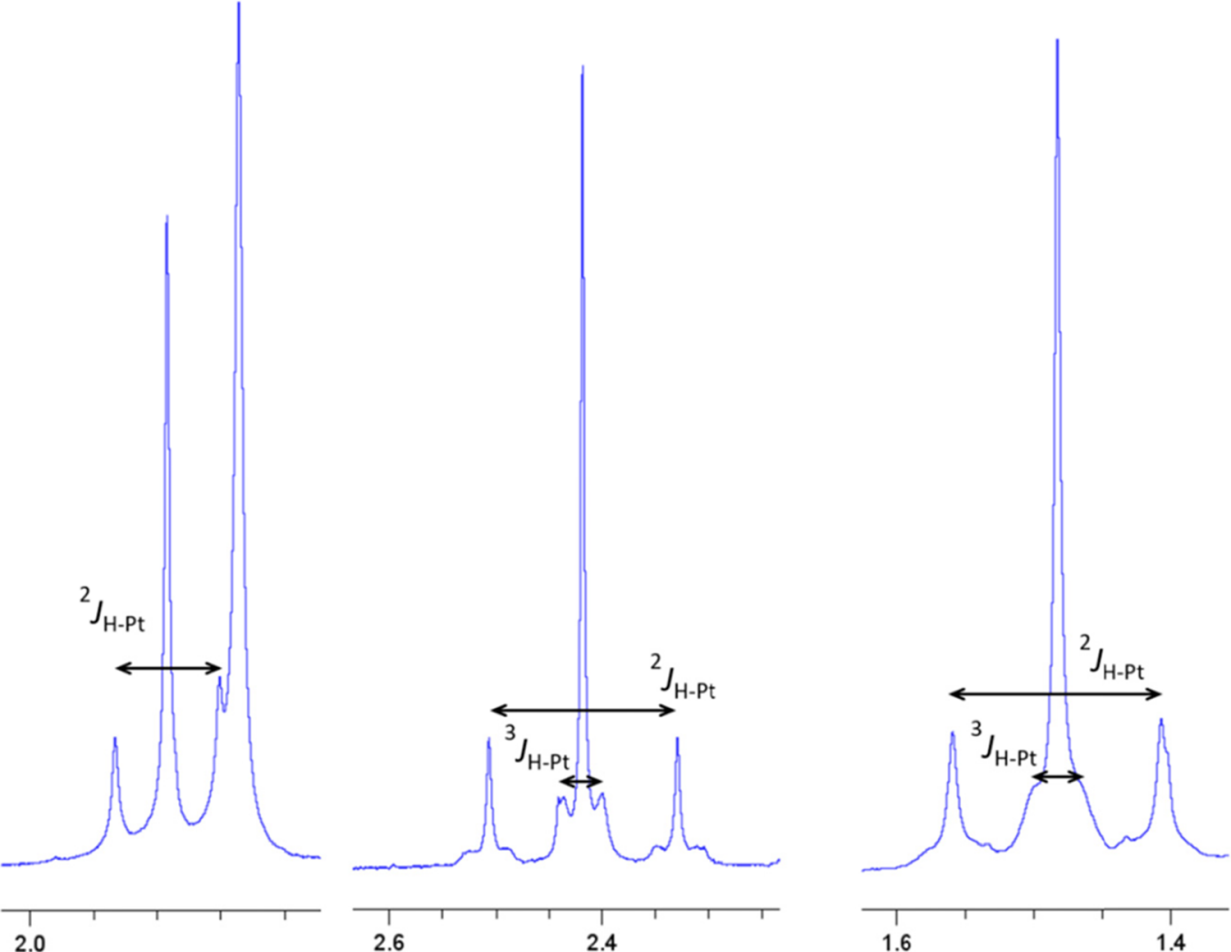
Expanded view of the ^1^H NMR spectra of **2a** (left), **3a′** (middle), and **4a** (right) in CD_2_Cl_2_.

**Figure 4:**
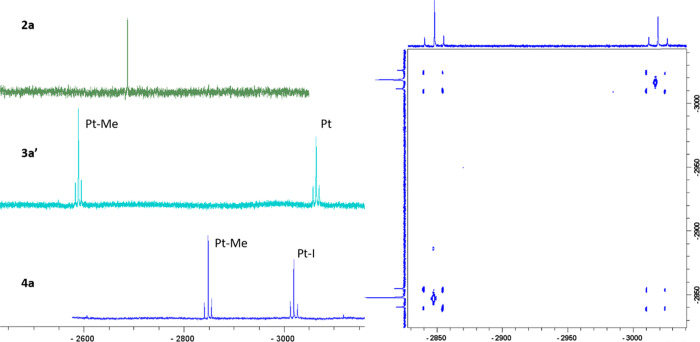
Left: ^195^Pt{^1^H} NMR spectra in CD_2_Cl_2_ of **2a**, **3a′**, and **4a**. Right: ^195^Pt–^195^Pt{^1^H} COSY spectrum of **4a** in CD_2_Cl_2_.

The NMR spectra of the Pt–Me derivatives, **2a**, **2b**, **3a′**, **4a**, and **Int′-Me**, as well as the Pt–Bn ones, **5a** and **6a**, were performed in CD_2_Cl_2_ solution (see Table 1 and Figures 3 and 4).

**Table 1:** Relevant NMR Data for the New High Oxidation State Pt_2_ Complexes^a^

compound	δ ^195^Pt–X	δ ^195^Pt–R	^1^*J*_Pt–Pt_	δ^1^H (Pt–R)	^2^*J*_Pt–H_	^3^*J*_Pt–H_
**2a**		–2688.0 (R = Me)		1.78	65.5	
**2b**		–2664.4^b^ (R = Me)		1.80	66.1	
**3a′**	–3064.2 X = vacant	–2589.2 (R = Me)	1023.4	2.42	70.7	14.7
**4a**	–3018.8 (X = I)	–2848.2 (R = Me)	1239.8	1.48	61.0	14.5
**Int′-Me**	–3776.0 (Pt^II^)	–2697.0 (Pt^IV^)		1.75	65.9	
**5a**	–2742.8 (X = Br)	–2693.6 (R = Bn)	1028.9	3.73 (1H)^c^	69.0	28.9
**6a**		–2357.0 (R = Bn)		4.02 (2H)^c^	90.6	

a CD_2_Cl_2_, more details in experimental Section, δ (ppm); *J* (Hz).

b Indirect detection by ^1^H–^195^Pt HMQC NMR.

c An equal signal appears overlapped with OC*H_2_*CH_3_; δ ^195^Pt **=** −3778.0 ppm (**1a**, acetone-d_6_), −3767.4 ppm (**1b**, DMSO-d_6_).

Their ^1^H NMR and ^195^Pt{^1^H} NMR spectra showed that in all cases, the major isomer is that observed in the X-ray single-crystal structures, with the C^C* groups in an *anti*-conformation, which provided structurally relevant details. In agreement with the absence of metal–metal interactions and their symmetry, the Pt_2_(IV,IV) complexes **2a**, **2b**, and **6a** show the coupling to just one ^195^Pt nucleus (see ^2^*J*_Pt-H_ in Table 1) of their corresponding Pt–R (R = CH_3_, CH_2_Ph) ^1^H NMR signals. Besides, their ^195^Pt{^1^H} NMR spectra exhibit just one singlet in the typical spectral range for Pt(IV) compounds (see Table 1 and Figure 4 for **2a**).^42^

By contrast, in compounds **3a′**, **4a**, and **5a**, the NMR spectra denote the non-equivalence of two Pt fragments joined by a metal–metal bond. That is, each compound exhibits a signal due to the Pt-CH_3_ (singlet) or Pt-CH_2_Ph (doublet) flanked by two sets of ^195^Pt satellites in its ^1^H NMR spectrum and two singlets in the ^195^Pt{^1^H} NMR one, each one flanked by ^195^Pt satellites.

The existence of a Pt–Pt bond was confirmed by a ^195^Pt–^195^Pt{^1^H} COSY spectrum, which displays a crosspeak due to scalar coupling (Figure 4 right for **4a** and S6 for **3a′**).

The assignment of these resonances was made from ^1^H–^195^Pt HMQC and ^1^H {selective^195^Pt} NMR experiments.

All the ^195^Pt signals appear clearly downfield-shifted with respect to those of the Pt_2_(II,II) complexes, **1a** and **1b** (Table 1), according to the higher oxidation state of the metal centers. They appear more deshielded as the oxidation state is higher [see δ^195^Pt for Pt^IV^-CH_3_ (**2a**) and Pt^IV^-CH_2_Ph (**6a**) vs Pt^III^-CH_3_ (**4a**) and Pt^III^-CH_2_Ph (**5a**)], and the electronegativity of the axial ligand is greater [see δ^195^Pt for Pt–Br (**5a**) vs Pt–I (**4a**)]. Besides, a downfield shift of the ^195^Pt-CH_2_Ph resonances with respect to the ^195^Pt–Me one is observed (see δ^195^Pt for **6a** vs **2a**), which is attributed to the effect of the π system of the benzyl fragment.^43^

The proposed structure for complex **Int′-Me** was based on its NMR data. The presence of two singlets in the spectral range expected for Pt^II^ and Pt^IV^ and the absence of platinum satellites in its ^195^Pt{^1^H} NMR spectrum denote the mixed-valence nature of **Int′-Me** and the absence of a metal–metal bond between the platinum centers. This fact was confirmed by its ^1^H NMR spectrum, which shows only one singlet corresponding to the Pt–Me group flanked just by one set of platinum satellites (Table 1, Figure S10).

The complex cation in **3a′** deserves some additional attention. In this complex, the average oxidation number of the platinum centers is +3, but it can be regarded as a metal–metal bonded Pt_2_(III,III) complex with just one axial ligand, or as a mixed valence Pt_2_(II,IV) one^44^ with the metals linked by a Pt^II^ → Pt^IV^ donor–acceptor bond. The short intermetallic distance (2.6700(4) Å) observed in the X-ray structure points to a Pt_2_(III,III) formulation, while the NMR data (Figure S7) point to a Pt^II^ → Pt^IV^ one. In this sense, the different coordination environments of the Pt centers cause a big separation between the two ^195^Pt resonances up to 480 ppm. The one corresponding to Pt–Me appears even more deshielded than that in the Pt_2_(IV,IV) compounds (**2a** and **2b**), while the other is shielded 50 ppm with respect to the Pt–I resonance in the Pt_2_(III,III) complex **4a**. To help determine the correct oxidation states of the Pt centers, we performed additional computational and electrochemical studies. The Mulliken population analysis in MeCN for **3a′** provided an estimated partial charge for the two platinum centers (0.49 Pt, 0.46 Pt–Me) not much different one to another, the difference (Δ = 0.03) being even lower than in the Pt_2_(III,III) complex, **4a** (0.35 Pt–I, 0.40 Pt–Me, Δ = 0.05). The Pt–Pt MO bond order in **3a′** (0.38) is close to the calculated value for the Pt_2_(II,II) complex **1a** (0.39) and smaller than that found for the Pt_2_(III,III) complex **4a** (0.59). These calculations are consistent with a Pt_2_(III,III) ↔ Pt_2_(II,IV) formulations, the contribution of the Pt_2_(II,IV) one being significant, in line with earlier calculations on catalytic processes involving [{Pd^III^(C^N)(OAc)}_2_XY]. They showed that when a strong σ-donor group is “axially” coordinated to one of the metal centers in dinuclear complexes, the dz^2^ orbital from the other metal gets populated, increasing the M(II) character and favoring the Pt_2_(II,IV) formulation.^45^ Therefore, in our case, the presence of Me as the electron-donating group in **3a′** would increase the Pt_2_(II,IV) contribution to this molecule.

In this sense, oxidative CV in MeCN showed for **3a′** an irreversible oxidation at 0.39 V (given vs the Fc+/Fc couple). The value is quite similar to that of **1a**, 0.44 V, measured under the same conditions and to the related cyclometalated pyrazolate-bridged dinuclear platinum(II) complexes,^46^ while being far from the value observed for the Pt_2_(III,III) complex [{Pt(C^C*_A_)(μ-pz)I}_2_] (Figure S14). In the Pt_2_(II,II) complexes, the irreversibility of this oxidation process has been attributed to the square-planar geometry of each Pt(II) unit with little or no metal–metal interaction. In them, the metal centers are highly susceptible to nucleophilic attack by coordinating solvents, such as MeCN, resulting in permanent oxidized products. Therefore, a mixed-valence Pt_2_(II,IV) formulation with the metals linked by a Pt^II^ → Pt^IV^ donor–acceptor bond seems to be the most likely for **3a′** in MeCN solution, which is also compatible with the observed metal–metal coupling.^47^ According to that, we observed that compound **3a′** reacted with MeI in acetonitrile to give **2a′**.

## Conclusions

Compound [{Pt^II^(C^C*_A_)(μ-pz)}_2_] (**1a**) reacted with MeI and BnBr at room temperature in the dark to give the high-valent dinuclear complexes [{Pt^IV^(C^C*_A_)Me(μ-pz)}_2_(μ-I)]I (**2a**) and [Br(C^C*_A_)Pt^III^(μ-pz)_2_Pt^III^(C^C*_A_)Bn] (**5a**), resulting from the double or single OA of RX, respectively. Also, [{Pt^II^(C^C*_B_)(μ-pz)}_2_] (**1b**) reacted with MeI, affording [{Pt^IV^(C^C*_B_)Me(μ-pz)}_2_(μ-I)]I (**2b**), indicating that the CO_2_Et substituent in C^C*_A_ does not affect the redox behavior of these Pt_2_(II,II) compounds **1a** and **1b**. DFT modeling of the S_N_2 mechanisms for the OA of RX to **1a** proposed species such as [(C^C*_A_)Pt(μ-pz)_2_Pt(C^C*_A_)R]X (RX = MeI **Int-Me**, BnBr **Int-Bn**) as intermediates for the first OA reaction. Once formed, two species are accessible, [X(C^C*_A_)Pt^III^(μ-pz)_2_Pt^III^(C^C*_A_)R](RX = MeI **4a**, BnBr **5a**) and [(C^C*_A_)Pt^II^(μ-pz)_2_Pt^IV^(C^C*_A_)(R)X] (RX = MeI **Int′-Me**, BnBr **Int′-Bn**), the latter being the intermediate for the second OA. Keeping in mind the small energy barrier for the transformation of **Int-R** into **Int′-R**, the free energy difference between the species Pt_2_(III,III) (**4a** or **5a**) and Pt_2_(II,IV) (**Int′-Me**, **Int′-Bn**) seems to determine the nature of the compounds obtained at r.t. When it is small (Δ*G*_Int′-Me-4a_ = 4.03 kcal/mol), the feasible formation of **Int′-Me** allows the second OA to occur, providing the Pt_2_(IV,IV) complex, **2a**. When it is bigger (Δ*G*_Int′-Bn-5a_ = 7.83 kcal/mol), the reaction leads to the selective formation of the Pt_2_(III,III) complex **5a**. In this case, the second OA to get [{Pt^IV^(C^C*_A_)Bn(μ-pz)}_2_(μ-Br)]Br (**6a**) is possible under harder conditions. Species **Int-Me** could be prepared and isolated as the BF_4_ salt, **3a′**, and then used to get **4a**, **Int′-Me**, and **2a′**, which indicate this computed mechanism as the most likely one and allow us to compare structural and spectroscopic data for complexes with the same core [{Pt(C^C*)(μ-pz)}_2_] but different oxidation states.

## Supporting Information Available

Information about general procedures and materials; X-ray structure determinations (CCDC 2160386–2160389); crystallographic data; DFT and electrochemistry methods; and multinuclear and NMR spectra and CV for characterization and mechanism’s determination and selected bond angles and lengths in the X-ray structures of **2a**, **2b**, **3a′**, and **5a** (PDF)
