# Supplementary material for: High-Valent Pyrazolate-Bridged Platinum Complexes: A Joint Experimental and Theoretical Study
Source: Inorg Chem. 2022 Aug 3;61(32):12559–69. doi: 10.1021/acs.inorgchem.2c01441 (PMC9387385; doi:10.1021/acs.inorgchem.2c01441)
Supplement: Supplementary file 1 — ic2c01441_si_001.pdf [file ic2c01441_si_001.pdf]

Supporting Information

High Valent Pyrazolate-Bridged Platinum Complexes: A Joint Experimental and Theoretical Study

Lorenzo Arnal, Daniel Escudero,* Sara Fuertes,* Antonio Martin and Violeta Sicilia*

[a]*Departamento de Quimica Inorganica, Facultad de Ciencias, Instituto de Sintesis Quimica y Catalisis Homogenea (ISQCH), CSIC - Universidad de Zaragoza, Pedro Cerbuna 12, 50009, Zaragoza (Spain). E-mail: sfuertes@unizar.es*

[b]*Department of Chemistry, KU Leuven, Celestijnenlaan 200f - box 2404, 3001 Leuven. E-mail: daniel.escudero@kuleuven.be*

[c]*Departamento de Quimica Inorganica, Escuela de Ingenieria y Arquitectura de Zaragoza, Instituto de Sintesis Quimica y Catalisis Homogenea (ISQCH), CSIC - Universidad de Zaragoza, Campus Rio Ebro, Edificio Torres Quevedo, 50018, Zaragoza (Spain)*. E-mail: sicilia@unizar.es

CONTENTS **Page**

1. Experimental section: S2

1.1 General procedures and materials S2

1.2 Crystallographic data (Table S1) S2

1.3 Computational Methods (Figure S1) S4

1.4 Electrochemistry S5

2. Results and discussion S6

2.1. Multinuclear NMR spectra and CV for characterization and mechanism's determination (Figures S2-S14) S6

2.2. Bonding parameters of the X-ray structures S27

3. References S28

1.-Experimental Section.

1.1. General procedures and materials.

IR spectra were recorded on a Perkin-Elmer Spectrum 100 FT-IR Spectrometer (ATR in the range 250-4000 cm^{-1}). Mass spectral analyses were performed with a Microflex MALDI-TOF Bruker or an Autoflex III MALDI-TOF Bruker instruments. C, H, and N analyses were carried out in a Perkin-Elmer 2400 CHNS analyzer. ^{1}H, ^{19}F, $^{13}C\{^{1}H\}$, $^{195}Pt\{^{1}H\}$ NMR spectra were recorded on Bruker Avance 400 MHz instrument using the standard references: $SiMe_4$ for ^{1}H and ^{13}C, $CFCl_3$ for ^{19}F and Na_2PtCl_6 in D_2O for ^{195}Pt. *J* is given in Hz; assignments are based on ^{1}H-^{1}H COSY, ^{1}H-^{13}C HSQC and HMBC, ^{1}H-^{195}Pt HMQC and ^{195}Pt-$^{195}Pt\{^{1}H\}$COSY experiments.

1.2. Crystallographic data.

Crystal data and details of the structure analyses are presented in Table S1. Suitable crystals for X-ray diffraction studies of **2a** and **2b** were obtained by slow diffusion of *n*-hexane into a concentrated solution of acetone. For **3a’** and **5a**, they were prepared by slow evaporation of concentrated solutions of CH_2Cl_2 (**3a’**) and MeCN (**5a**). Crystals were mounted at the end of a quartz fibre. The radiation used in all cases was graphite monochromated MoK_{α} (λ = 0.71073 Å). X-ray intensity data were collected on an Oxford Diffraction Xcalibur diffractometer. The diffraction frames were integrated and corrected from absorption by using the CrysAlis RED program.[1] The structures were solved by Patterson and Fourier methods and refined by full-matrix least squares on F^2 with SHELXL.[2] All non-hydrogen atoms were assigned anisotropic displacement parameters. The positions of the hydrogen atoms were constrained to idealised geometries and assigned isotropic displacement parameters equal to 1.2 or 1.5 times the U_{iso} values of their respective parent atoms. Full-matrix least-squares refinement of the models against F^2 converged to final residual indices given in Table S1.

Table S1. Crystallographic data

	**2a·(CH$_3$)$_2$CO**	**2b·2 (CH$_3$)$_2$CO**	**3a'·2 CH$_2$Cl$_2$**	**5a·0.75·CH$_3$CN**
Empirical formula	$C_{37}H_{44}I_2N_8O_5Pt_2$	$C_{34}H_{42}I_2N_8O_5Pt_2$	$C_{35}H_{39}BCl_4F_4N_8O_4Pt_2$	$C_{40.50}H_{41.25}BrN_{8.75}O_4Pt_2$
Formula weight	1324.78	1238.73	1254.53	1184.66
Crystal system	Monoclinic	Monoclinic	Triclinic	Triclinic
temperature	100(2) K	100(2) K	100(2) K	100(2) K
Space group	C2/c	P 2/n	P -1	P -1
a (Å)	24.5751(10)	13.2136(2)	11.1281(3)	12.3207(5)
b (Å)	6.7647(3)	6.50316(12)	13.8750(4)	13.6167(6)
c (Å)	26.5176(11)	22.5852(4)	15.3399(5)	14.1184(7)
α (°)	90	90	104.095(3)	95.756(4)
β (°)	111.388(5)	103.6743(18)	92.568(2)	113.818(4)
γ (°)	90	90	113.306(3)	98.045(4)
Volume (Å3)/Z	4104.8(3)/4	1885.74(6)/2	2083.09(12)/2	2113.16(18)/2
ρ (Mg/m3)	2.144	2.182	2.000	1.862
μ (Mo-Kα)/mm-1	8.364	9.089	7.032	7.609
F(000)	2496	1160	1204	1137
Crystal size (mm3)	0.240 x 0.050 x 0.040	0.390 x 0.060 x 0.040	0.320 x 0.260 x 0.070	0.140 x 0.090 x 0.050
Theta range (°)	2.834 to 28.246	2.860 to 28.466	2.773 to 28.361	2.466 to 29.191
Reflections collected	17054	34612	29167	37270
Independent reflections [R(int)]	4406 [R(int) = 0.0420]	4382 [R(int) = 0.0550]	8808 [R(int) = 0.0510]	10111 [R(int) = 0.0898]
Final R1, wR2 [I>2sigma(I)]	0.0401, 0.1012	0.0303, 0.0698	0.0405, 0.0989	0.0578, 0.0947
R1,wR2 (all data)	0.0487, 0.1061	0.0352, 0.0734	0.0528, 0.1075	0.1096, 0.1095
GOF (F^2)	1.110	1.033	1.046	1.049
Largest diff. peak and hole/e.Å^{-3}	2.427 and -1.356	2.300 and -2.579	1.992 and -2.073	1.994 and -2.643

§ $wR2 = [\sum w\Delta^2 / \sum wF_o^4]^{0.5}$; $S = [\sum w\Delta^2 / (N - NV)]^{0.5}$; $R1 = \sum ||F_o| - |F_c|| / \sum |F_o|$; $\Delta = F_o^2 - F_c^2$; N = NO + restraints; $w = [\sigma_c^2(F_o^2)+(gP)^2]^{-1}$, $\sigma_c^2(F_o^2)$ = variance in F_o^2 due to counting statistics, $P = [\max(F_o^2,0)+2F_c^2]/3$.

1.3.-Computational methods

Density functional calculations were carried out on the ground (S_0) state with the Gaussian 16[3] suite of programs, using the M06 hybrid density functional[4] (MUE (M06) = 2.48 kcal/mol[5]) together with Grimme's D3 dispersion correction[6]. The ECP-60-mwb for platinum and ECP-46-mwb, for iodine, pseudopotential[7] was used, and the 6-31G(d)[8, 9] basis sets were used for all other atoms. In order to facilitate the theoretical study, we have done a simplification on the real system, we have modelled the ethanoate substituent on the cyclometalated ligand as an acetate. General geometry optimizations were performed without any symmetry restriction and in MeCN and Anisole by using the polarizable continuum model (PCM)[10]. Complexes **Int-R (R=Me, Bn)**, **2a** and **6a** were optimized as neutral species, cation plus anion. Frequency calculations were performed in order to determine the nature of the stationary points found in So (no imaginary frequencies for minima and only one imaginary frequency for **TS1 Me**(436i cm^{-1}), **TS1 Bn**(294i cm^{-1}), **TS2 Me**(43i cm^{-1}), **TS2 Bn**(44i cm^{-1}), **TS3 Me**(423icm^{-1}), **Bn**(260icm^{-1})). Mulliken population analysis was carried out as implemented in Gaussian 16 package[3]. ChemissianLab program package was used for analysis and graphic representation of molecular structures and orbitals and for Mayer Bond Order analysis. Atomic charges were calculated by using the NBO analysis option as incorporated in Gaussian 16.

Complexes **1a, TS1-R, Int-R, 4a, 5a, TS2-R, Int'-R** were modelled separately and their ΔG were summed with the ΔG of the required MeI modelled molecules, so that there is always the same number of atoms, those corresponding to **1a** +2MeI. The corresponding sum of these values is the represented in each case.

Atomic coordinates for the optimized structures are included as a separate .xyz file.

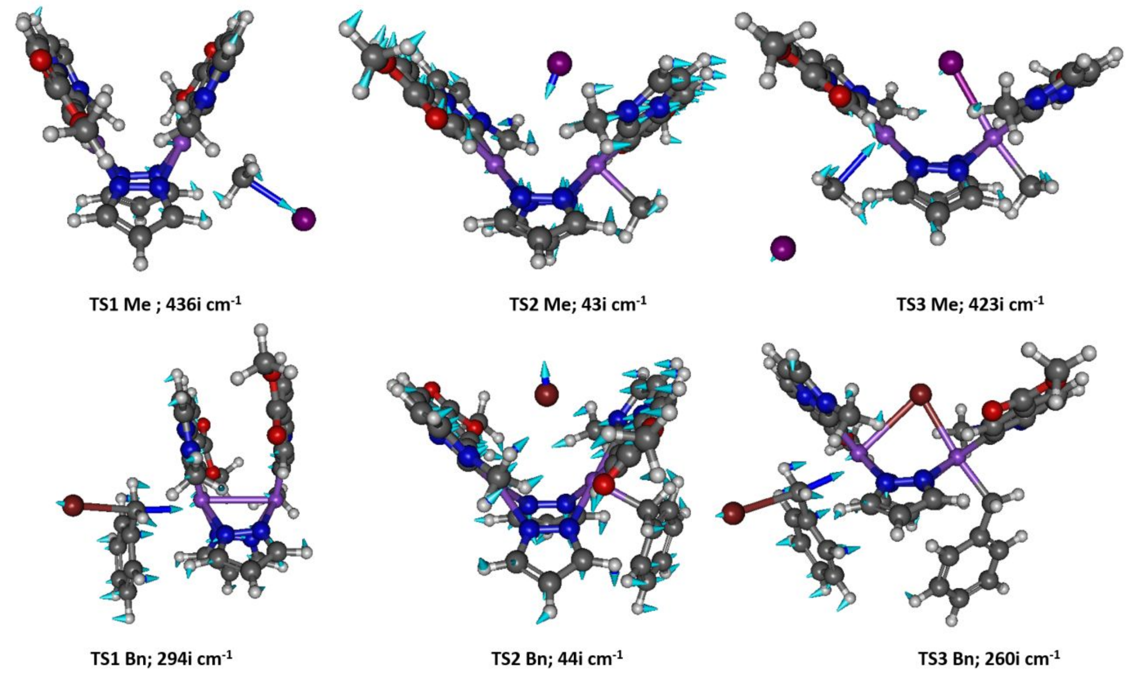

Figure S1. Gradient difference Vector for TS1-3 Me (top) and TS1-3 Bn (bottom)

1.4.-Electrochemistry

Cyclic voltammetry of **1a**, **3a'** and [Pt(C^C_A*)(μ-pz)I}$_2$] were performed using a VoltaLab PST050 electrochemistry workstation in a conventional three-electrode arrangement with all experiments performed in an O_2-free atmosphere. Oxidation potentials were measured in 5x10^{-4} M solutions in MeCN containing 0.1 M tetrabutylammonium hexafluorophosphate as the supporting electrolyte. A platinum wire was used as working electrode, a platinum wire as the counter electrode, and SCE as the reference electrode. Measurements were conducted with a scan rate of 100 mV/s, and ferrocenium/ferrocene (Fc+/Fc) was used as the internal standard.

2.- Results and discussion

2.1. Multinuclear NMR spectra for characterization

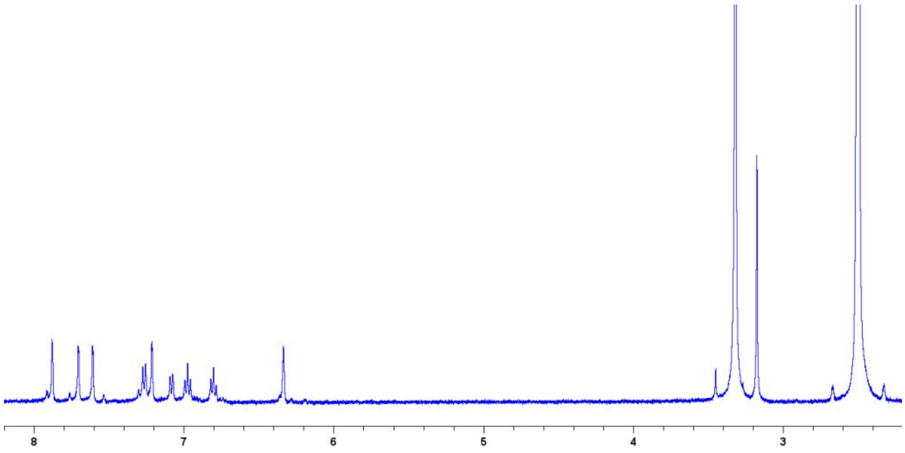

(a)

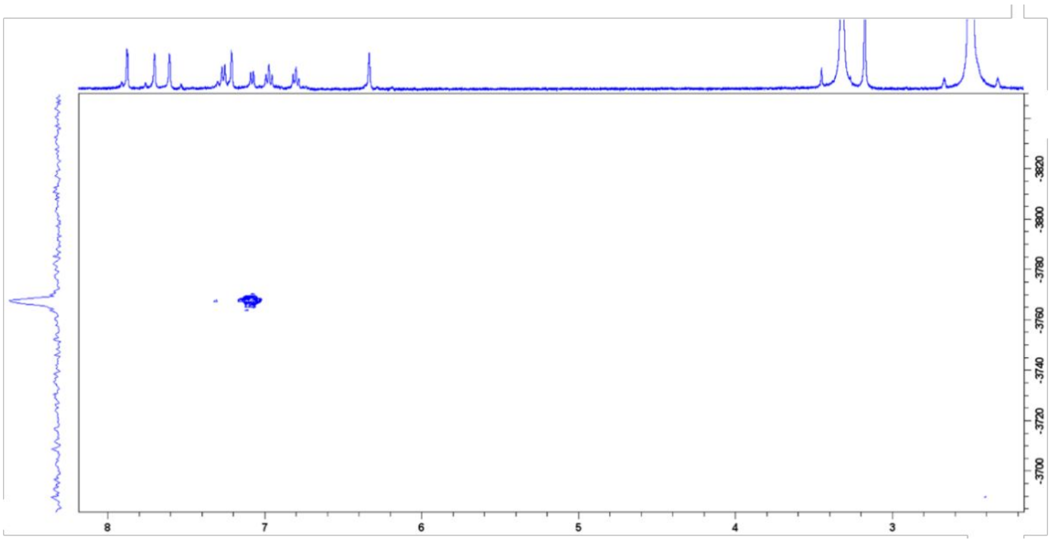

(b)

Figure S2. NMR Spectra of **1b** in DMSO- d_6. ^{1}H (a), ^{1}H-^{195}Pt HMQC ($J_{H\text{-}X}$=50Hz) (b).

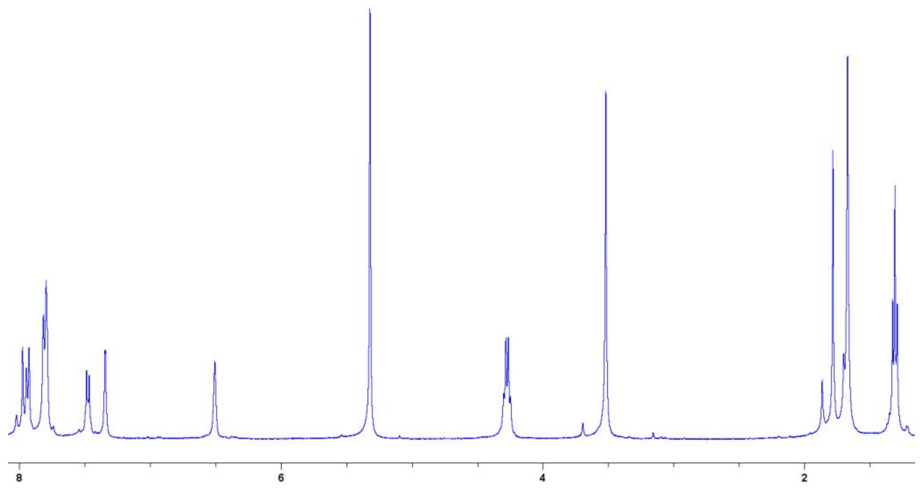

(a)

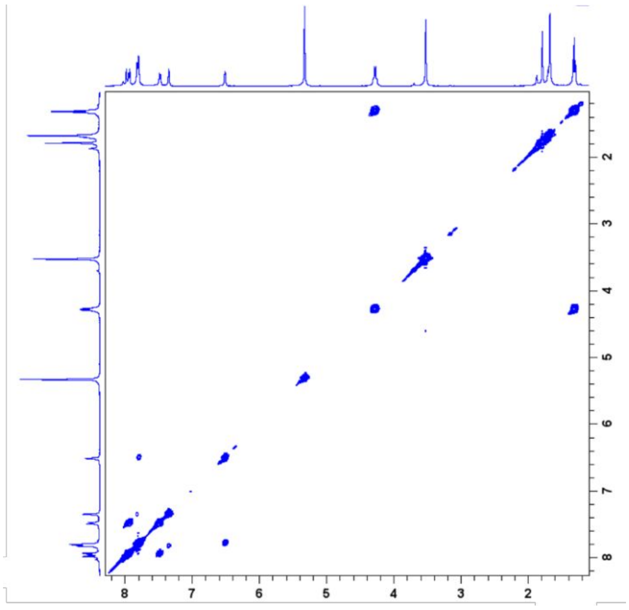

(b)

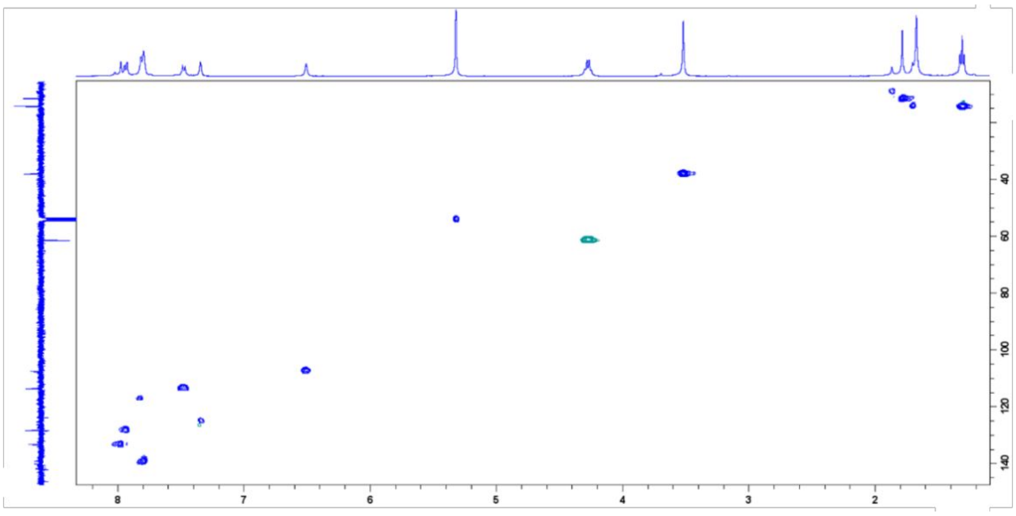

(c)

Figure S3a-c. NMR spectra of **2a** at 248K in methylene chloride- d_2. ^1H (a), ^1H-^1H COSY (b), ^1H-^{13}C HSQC (c).

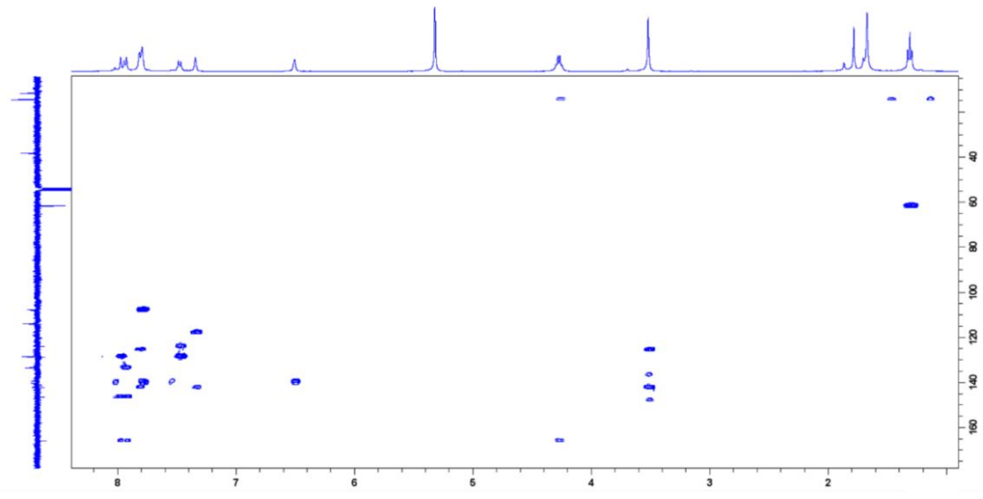

(d)

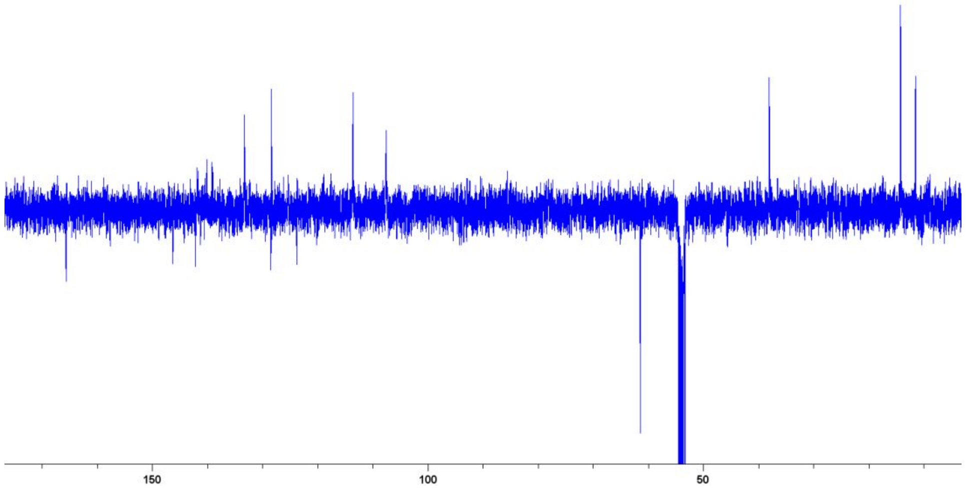

(e)

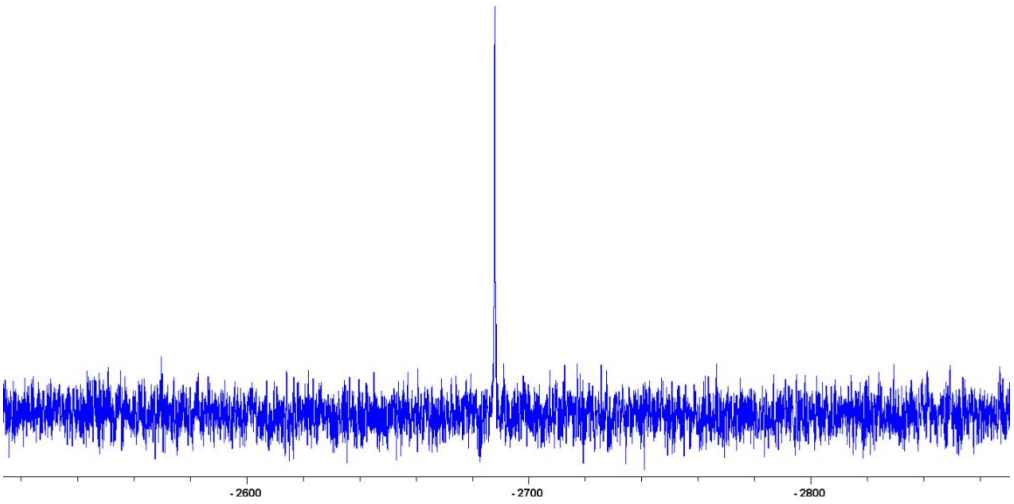

(f)

Figure S3d-f. NMR Spectra of **2a** at 248K in methylene chloride- d_2. ^{1}H-^{13}C HMBC (d), ^{13}C {^{1}H} APT (e), ^{195}Pt{^{1}H} (f).

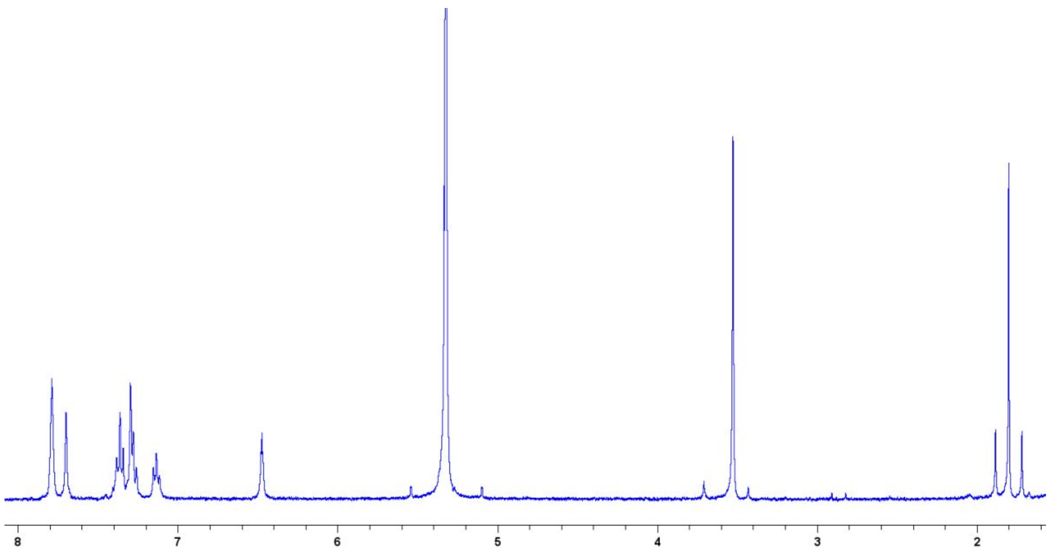

(a)

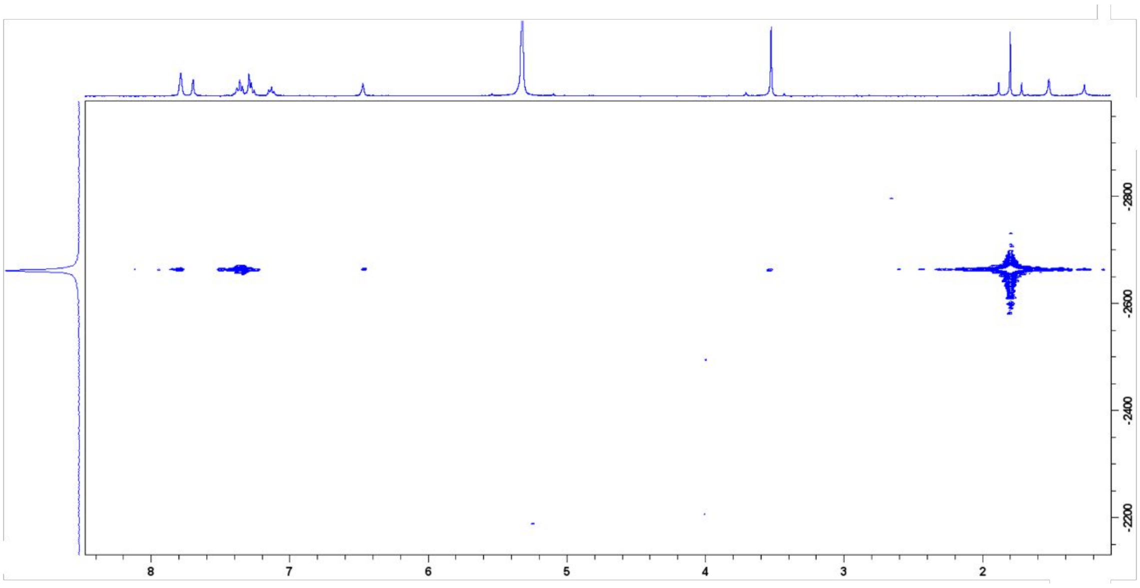

(b)

Figure S4. NMR Spectra of **2b** in methylene chloride- d_2. ^{1}H (a), ^{1}H-^{195}Pt HMQC (J_{H-X}=50Hz) (b).

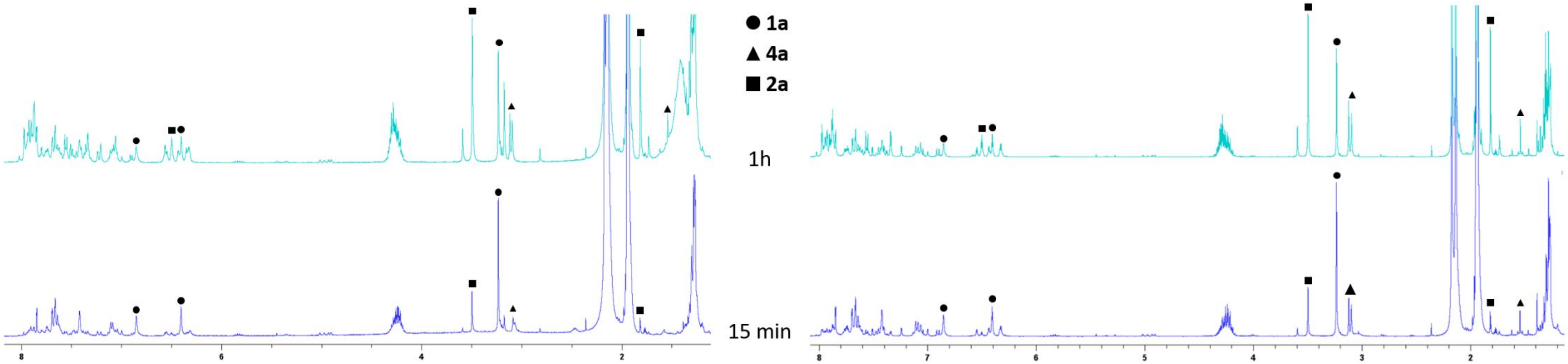

Figure S5. ^{1}H NMR spectra of the reaction of **1a + MeI** in MeCN-d_3 with Gal·(left) and without Gal·(right).

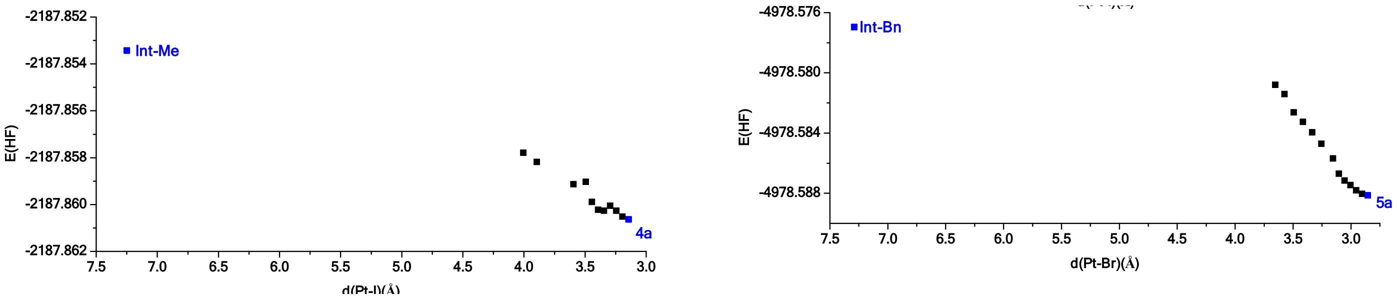

Figure S6. Relaxed Scans from **Int-R** to **4a/5a**

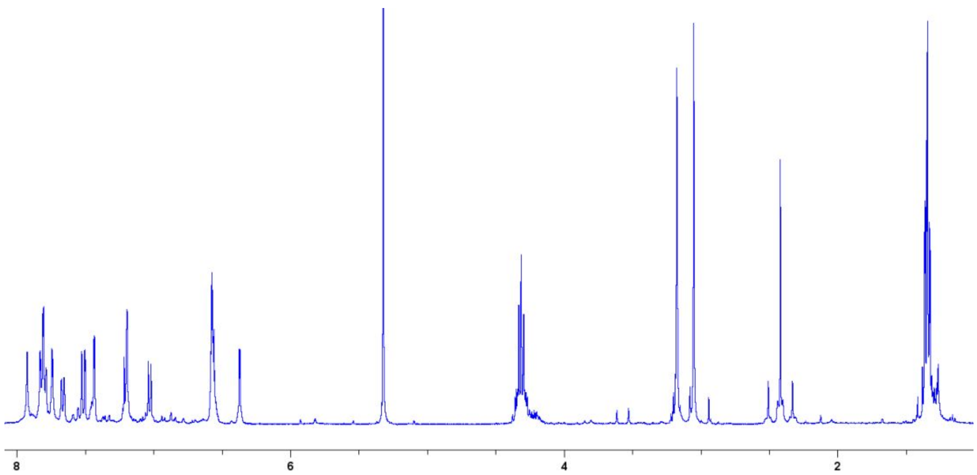

(a)

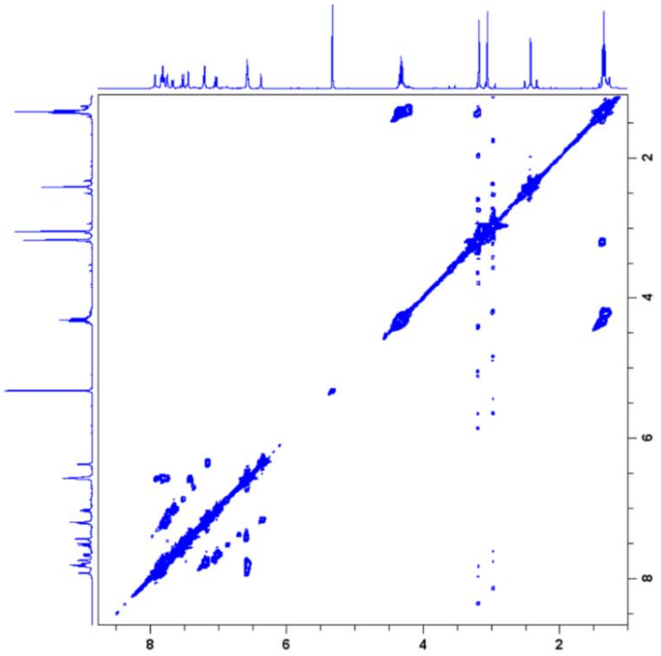

(b)

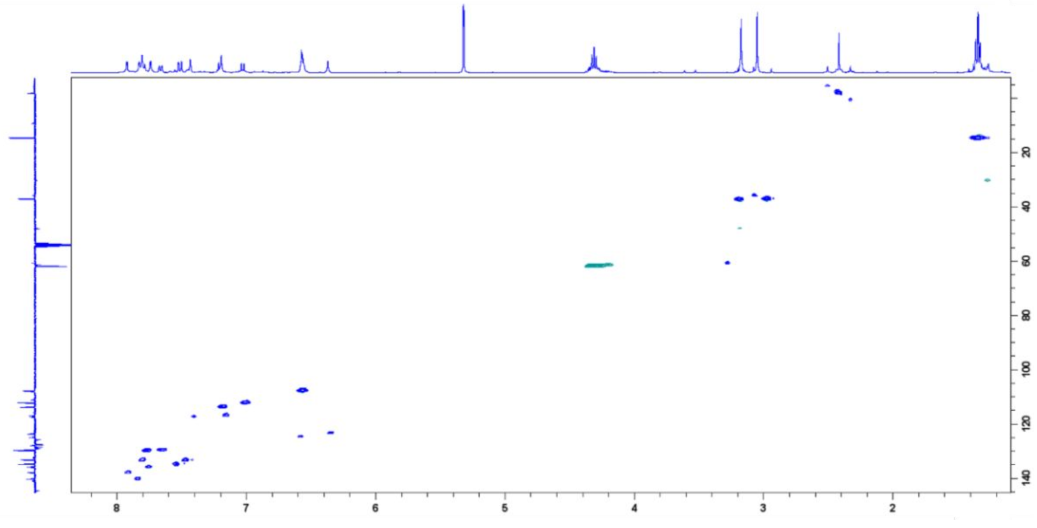

(c)

Figure S7a-c. NMR Spectra of **3a’** in methylene chloride- d_2. ^{1}H (a), ^{1}H-^{1}H COSY (b), ^{1}H-^{13}C HSQC (c),

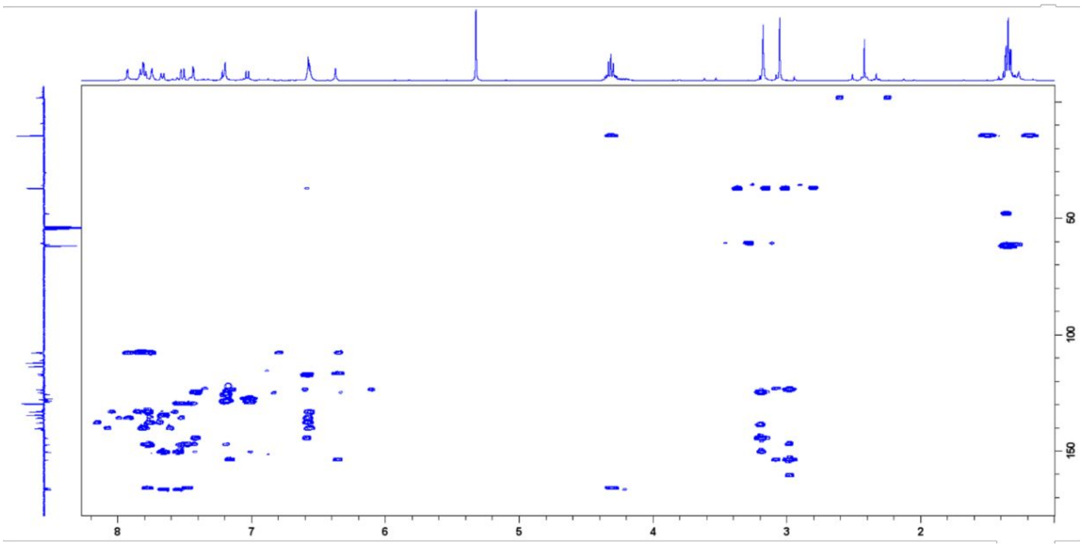

(d)

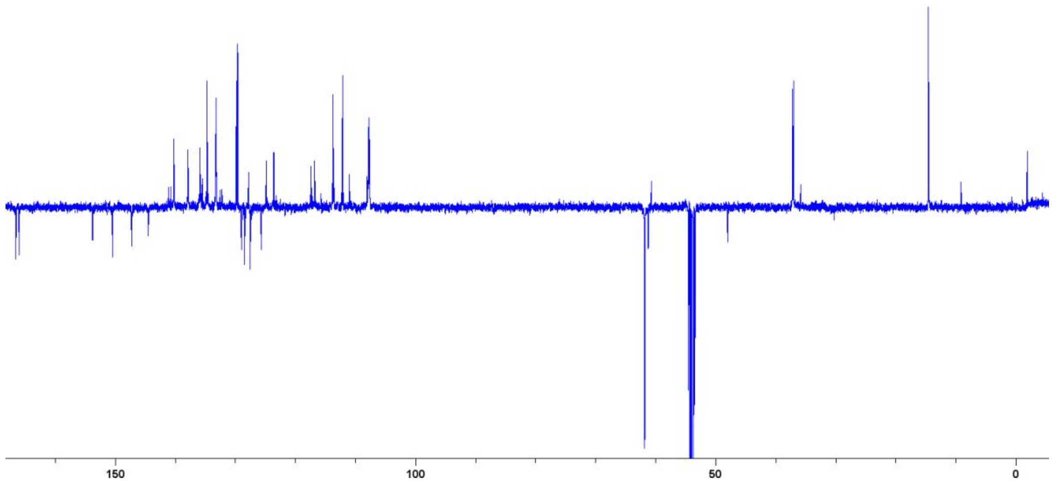

(e)

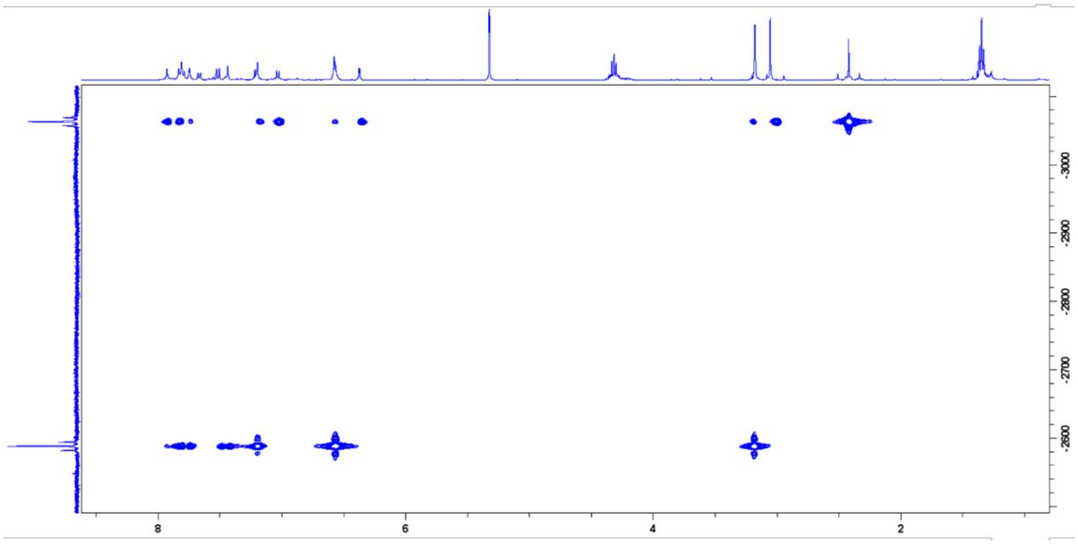

(f)

Figure S7d-f. NMR Spectra of **3a'** in methylene chloride- d_2. ^{1}H-^{13}C HMBC (d), ^{13}C $\{^{1}H\}$ APT (e), ^{1}H-^{195}Pt HMQC ($J_{H\text{-}X}$=12Hz) (f).

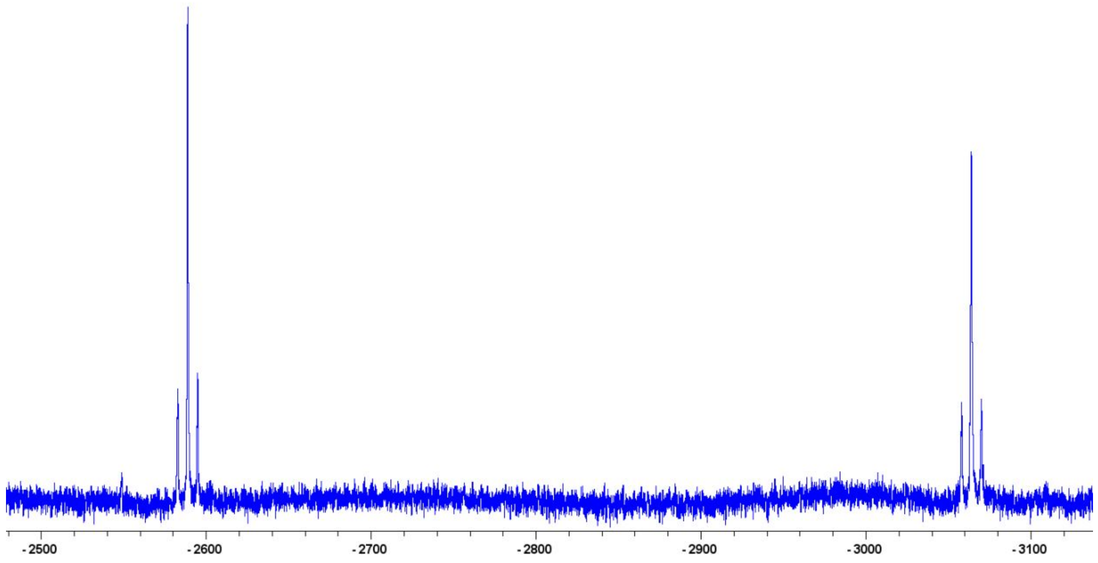

(g)

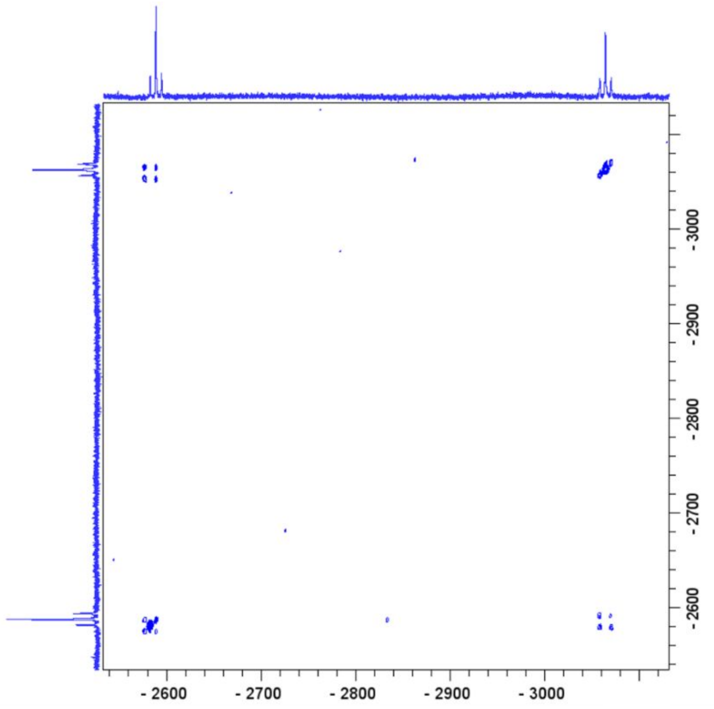

(h)

Figure S7g-h. NMR Spectra of **3a'** in methylene chloride- d_2. ^{195}Pt{^{1}H} (g), ^{195}Pt-^{195}Pt{^{1}H} COSY (h).

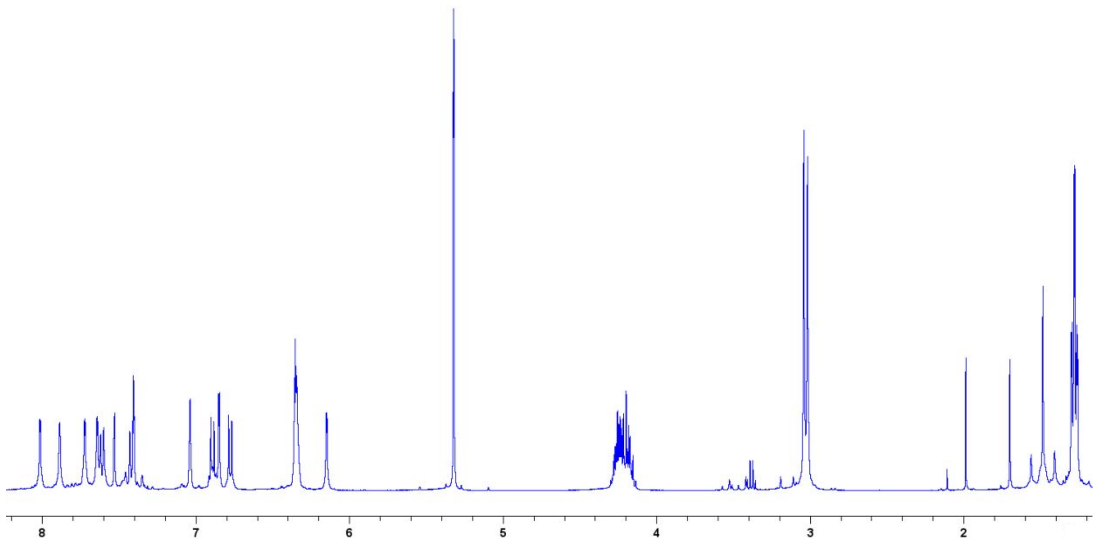

(a)

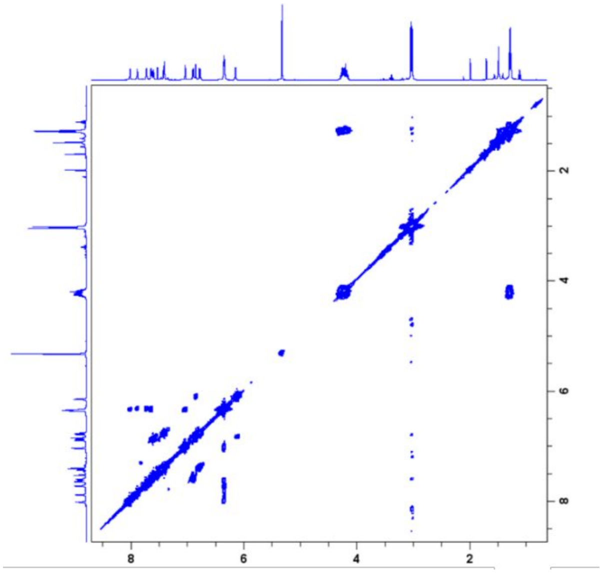

(b)

Figure S8a-b. NMR Spectra of **4a** at 223K in methylene chloride- d_2. ^1H (a), ^1H-^1H COSY (b),

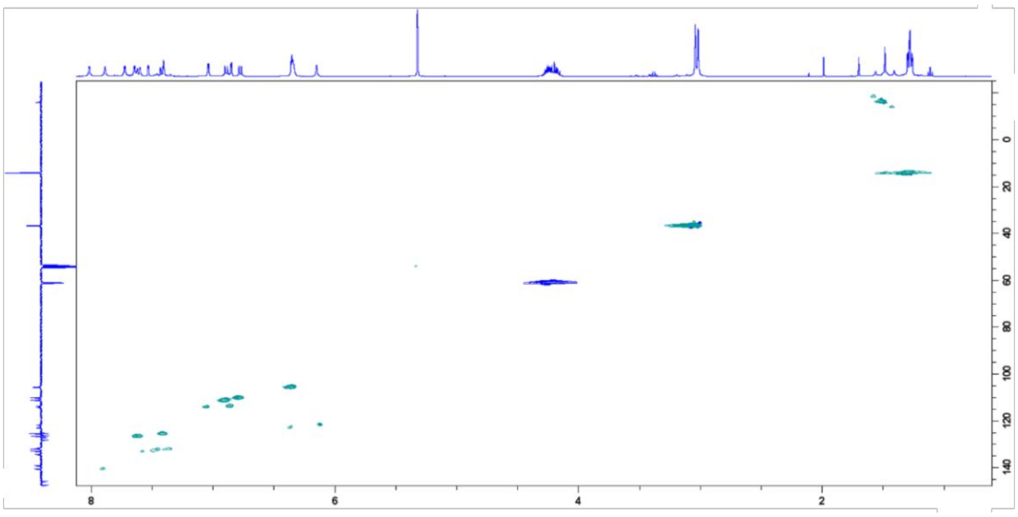

(c)

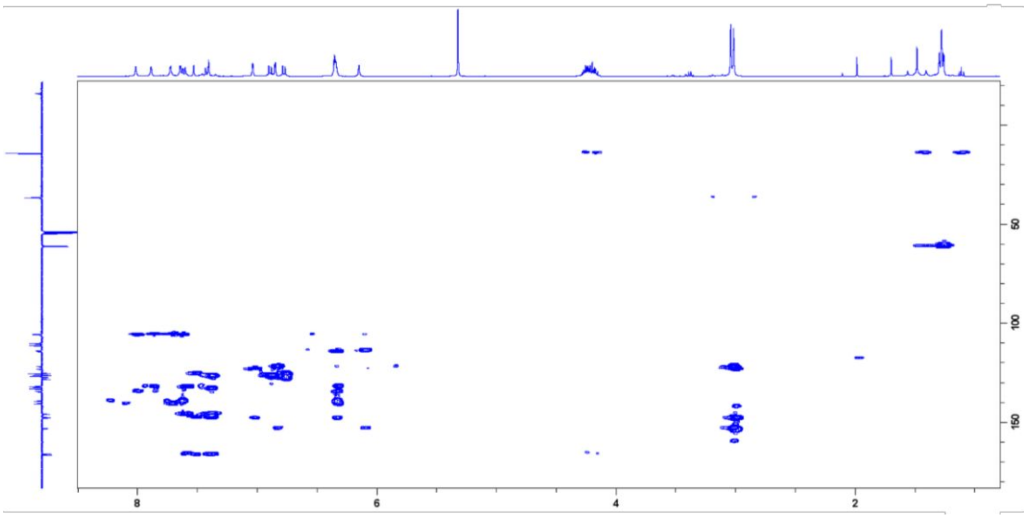

(d)

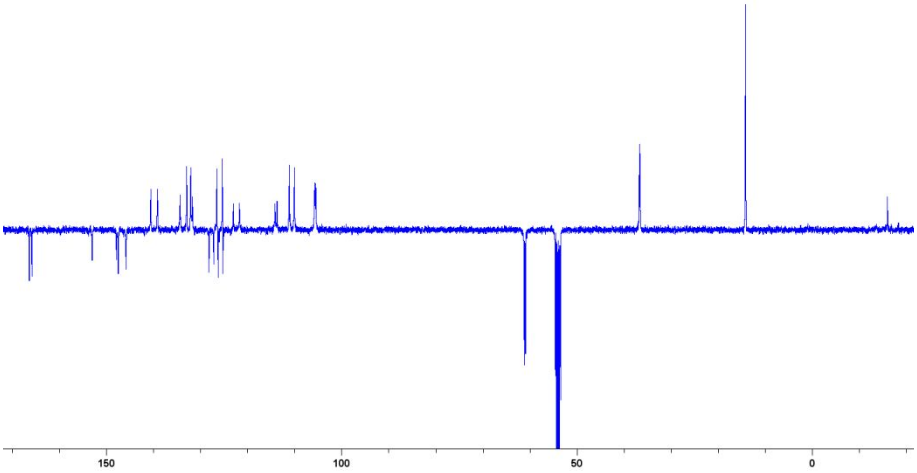

(e)

Figure S8c-e. NMR Spectra of **4a** at 223K in methylene chloride- d_2. ^1H-^{13}C HSQC (c), ^1H-^{13}C HMBC (d), ^{13}C {^1H} APT (e).

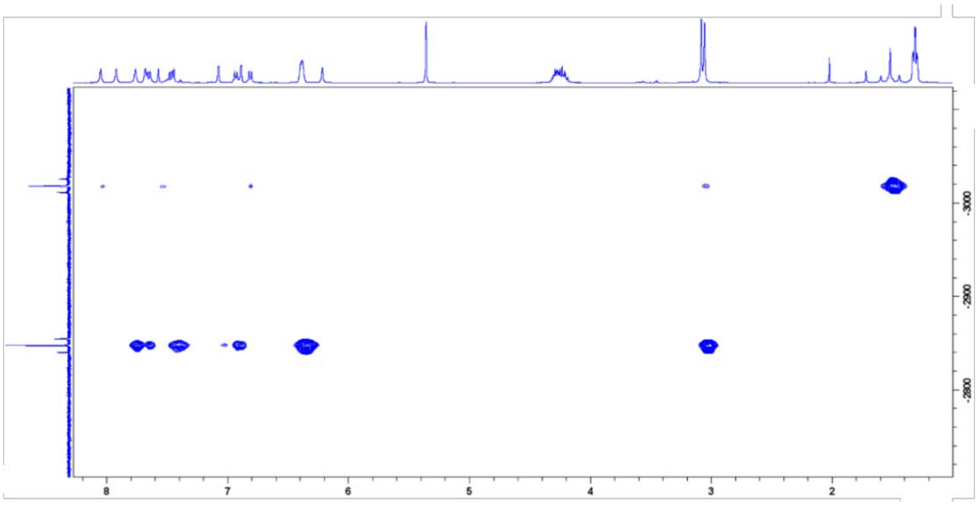

(f)

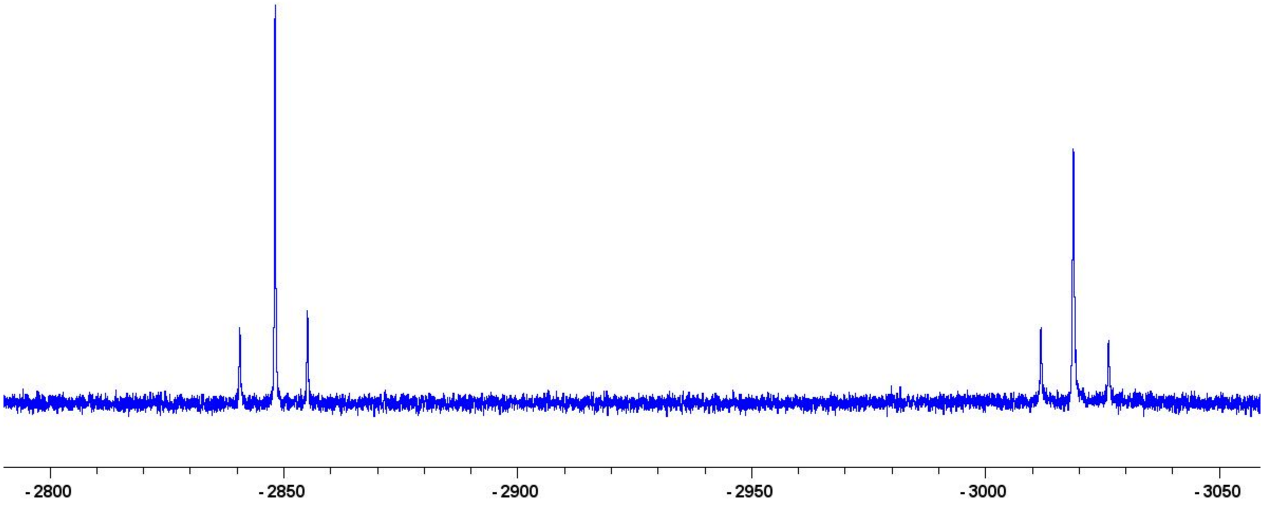

(g)

Figure S8f-g. NMR Spectra of **4a** at 223K in methylene chloride- d_2. ^{1}H-^{195}Pt HMQC ($J_{H\text{-}X}$=15Hz) (f), ^{195}Pt{^{1}H} (g).

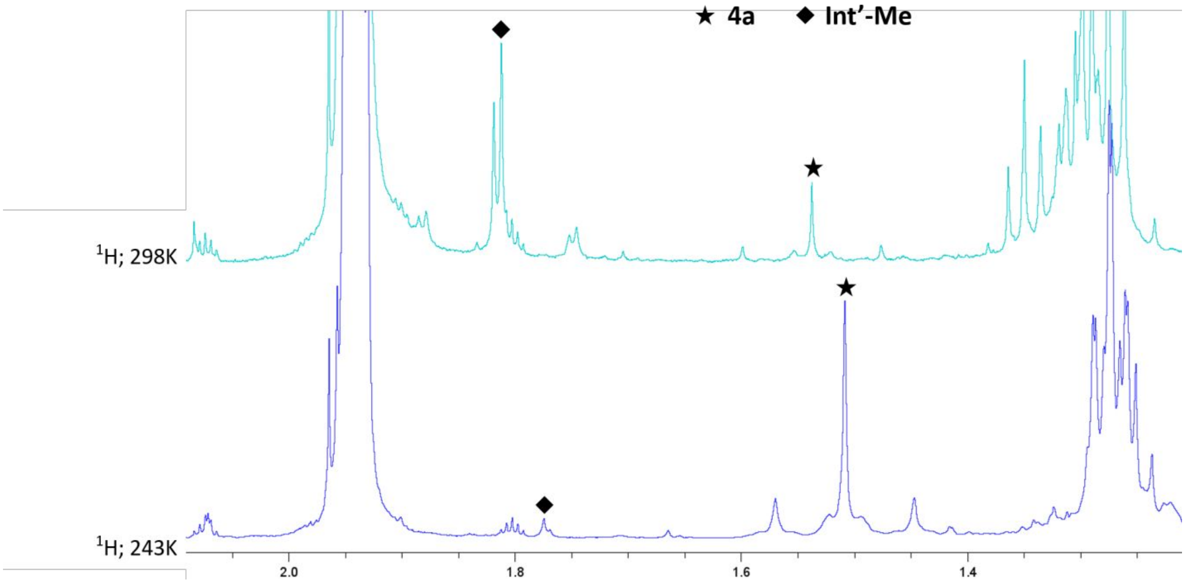

Figure S9: ^{1}H NMR spectra for the reaction of **3a'** with KI at -30ºC in NMR tube recorded at 243 K (bottom) and after 24 h at 298 K (top).

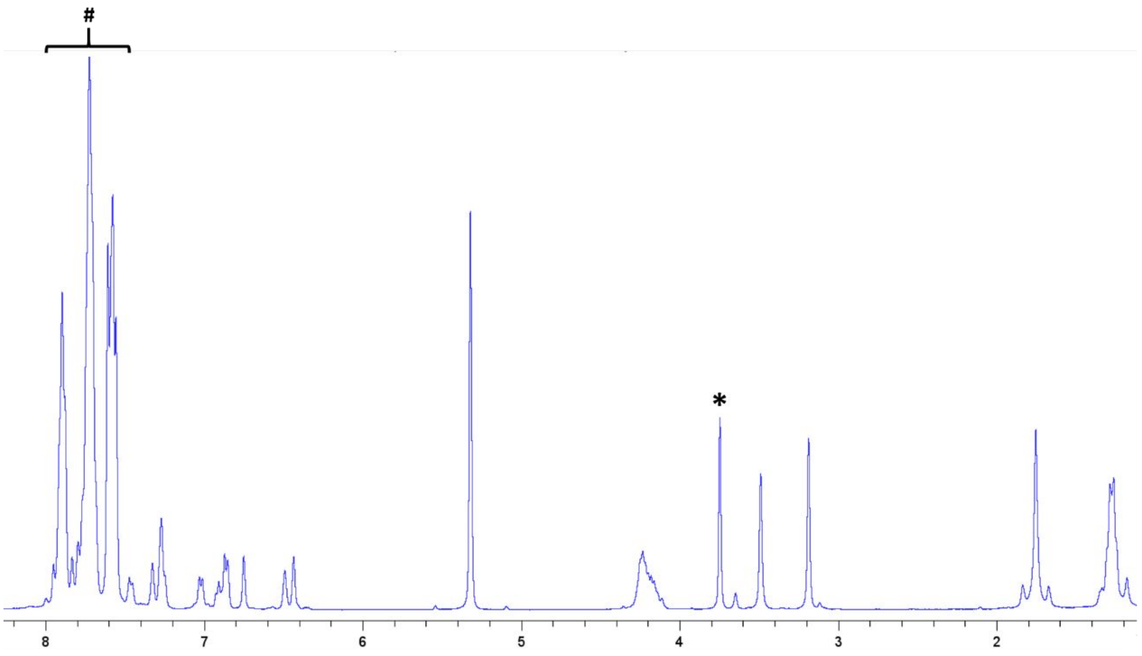

(a)

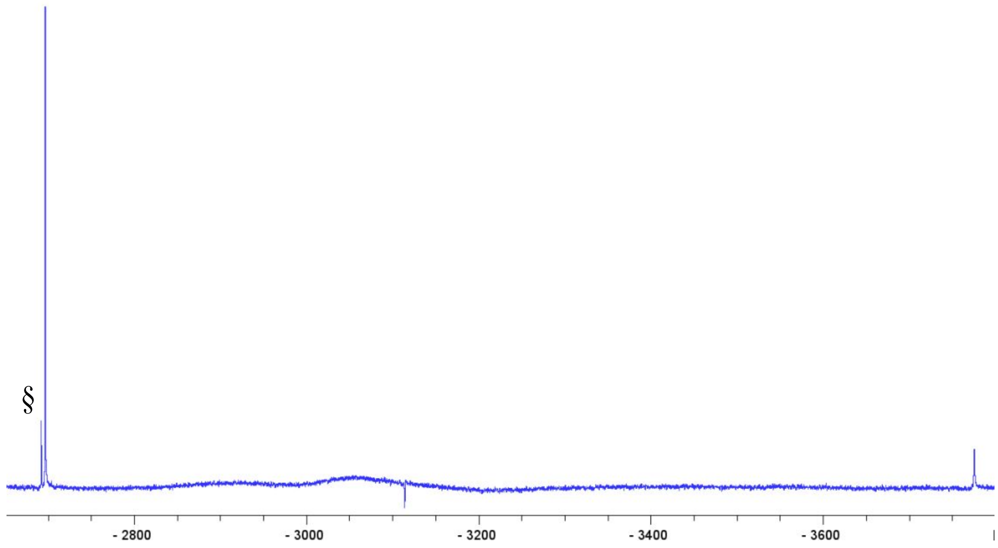

(b)

Figure S10. NMR Spectra of **Int'-Me** at 223K in methylene chloride- d_2. ^{1}H (a) (***Anisole, # PPh_4BF_4) ^{195}Pt{^{1}H} (b) (§: Pt^{IV} of the *syn*- **Int'-Me**).

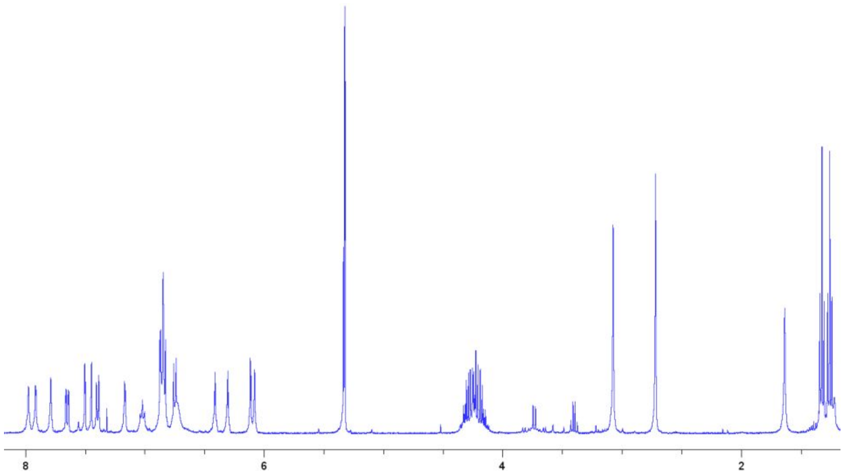

(a)

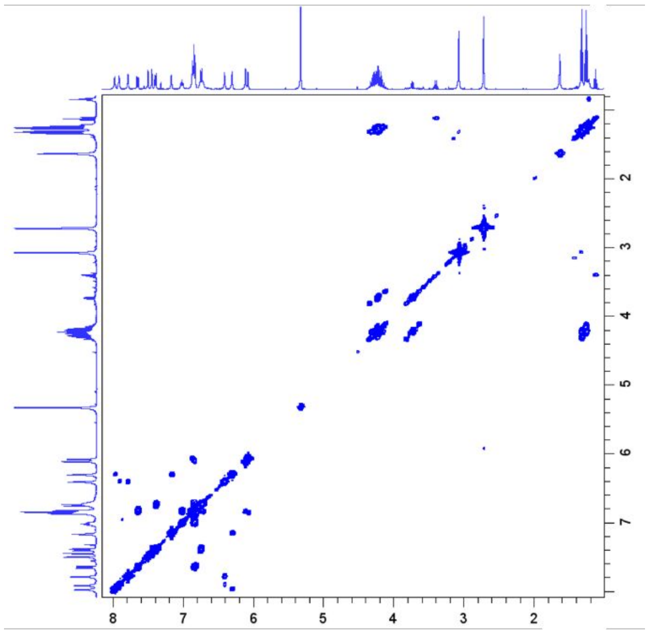

(b)

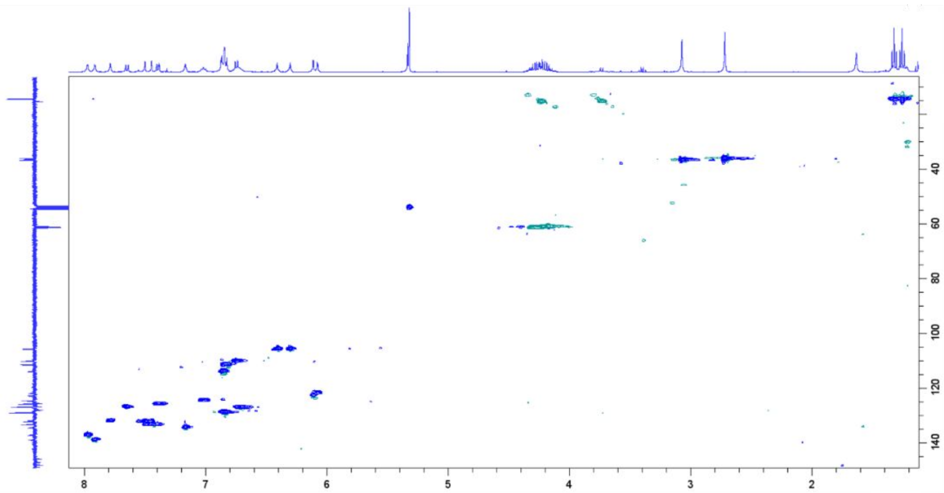

(c)

Figure S11a-c. NMR Spectra of **5a** at 248K in methylene chloride- d_2. ^{1}H (a), ^{1}H-^{1}H COSY (b), ^{1}H-^{13}C HSQC (c).

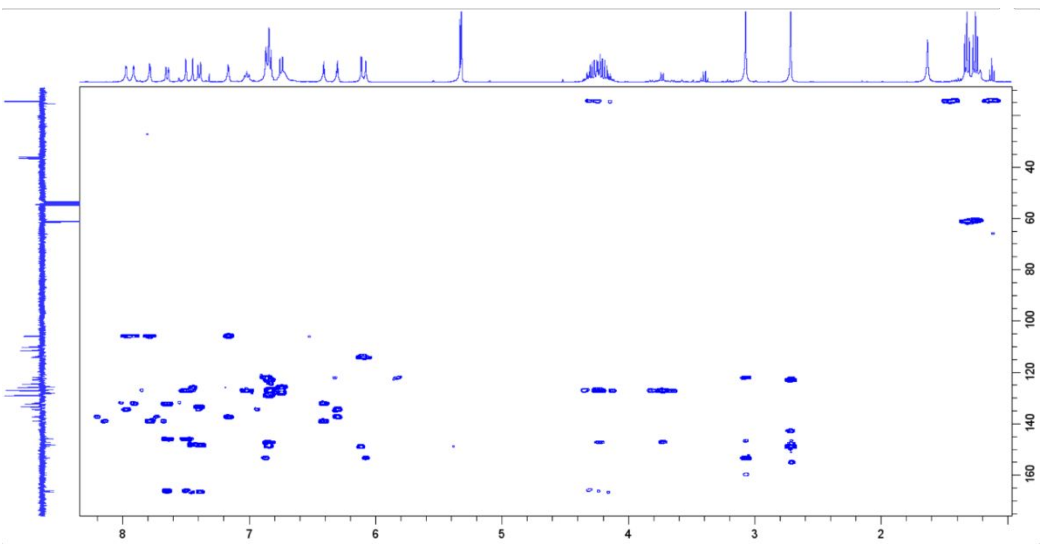

(d)

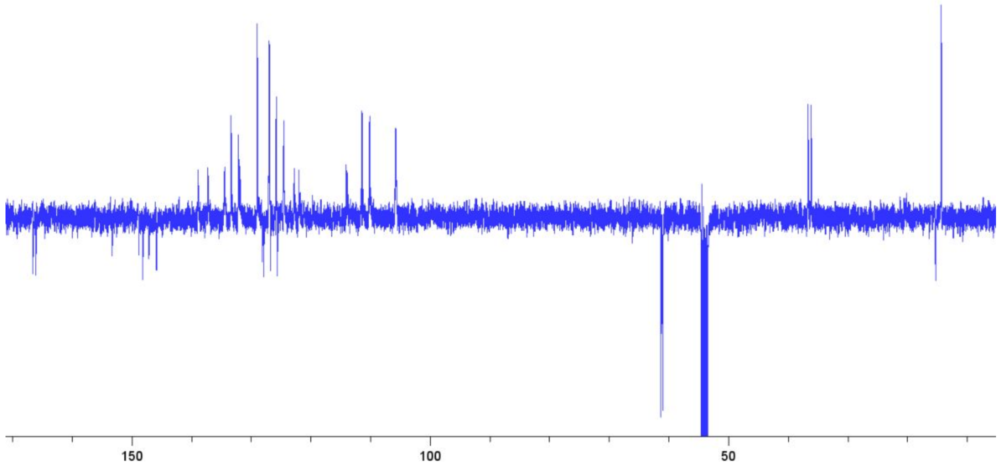

(e)

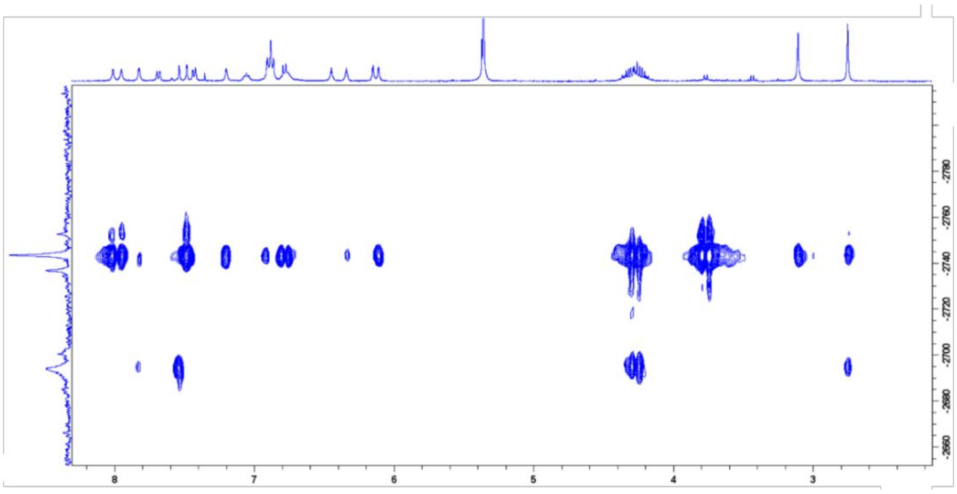

(f)

Figure S11d-f. NMR Spectra of **5a** at 248K in methylene chloride- d_2. ^1H-^{13}C HMBC (d), ^{13}C {^1H} APT (e), ^1H-^{195}Pt HMQC ($J_{H\text{-}X}$=30Hz) (f),

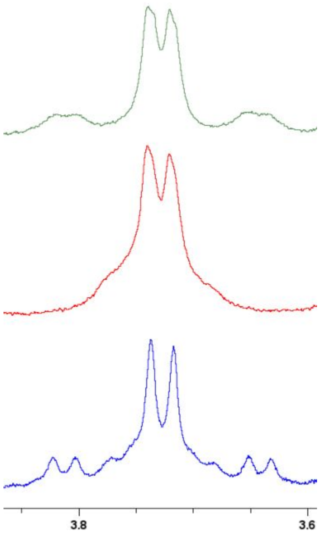

(g)

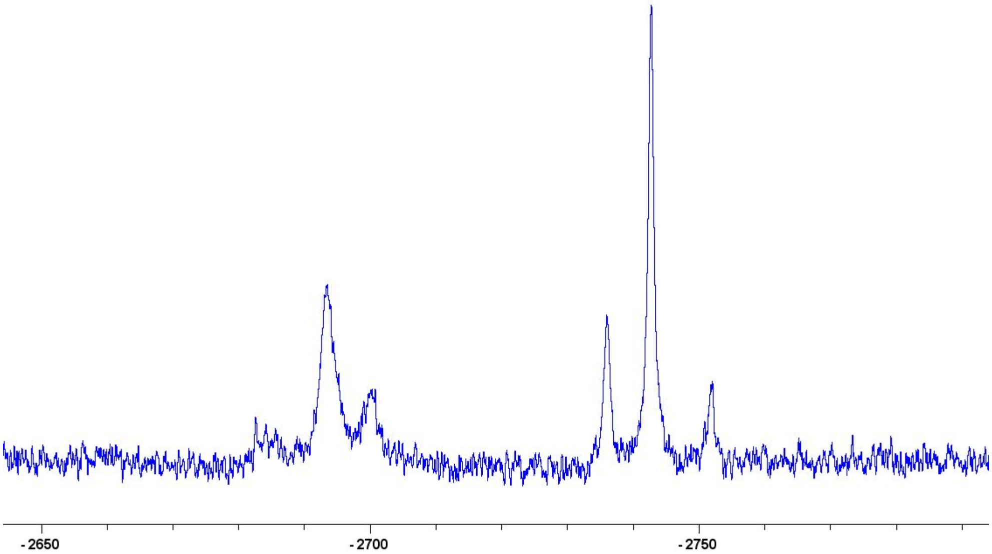

(h)

Figure S11g-h. NMR Spectra of **5a** at 248K in methylene chloride- d_2. ^{1}H (blue), ^{1}H{selective^{195}Pt}(δPt=-2693.6ppm) (red) and ^{1}H{selective^{195}Pt}(δPt=-2742.8ppm) (green), resonance of 3.73ppm (g), ^{195}Pt{^{1}H} (h).

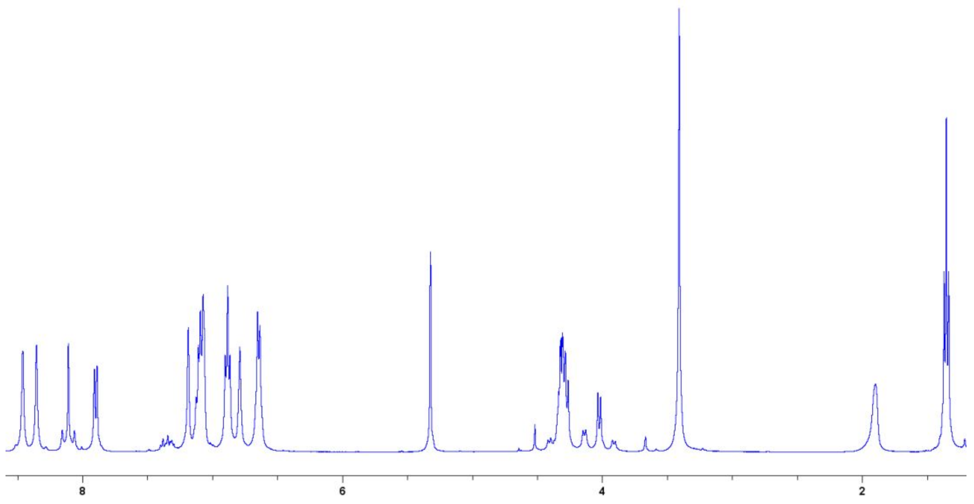

(a)

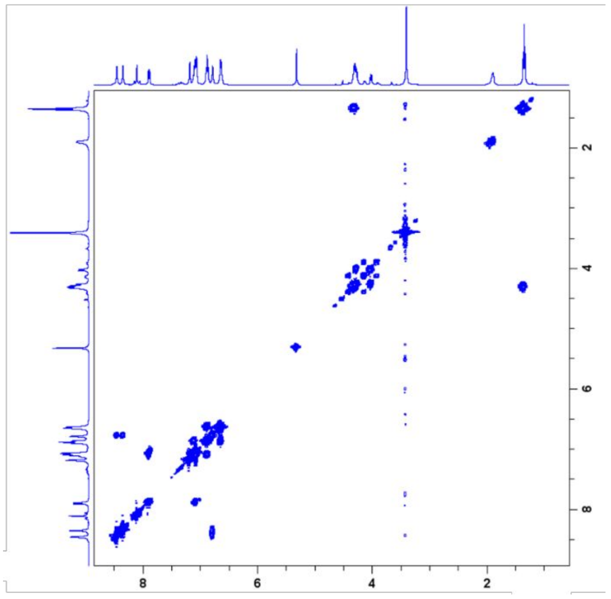

(b)

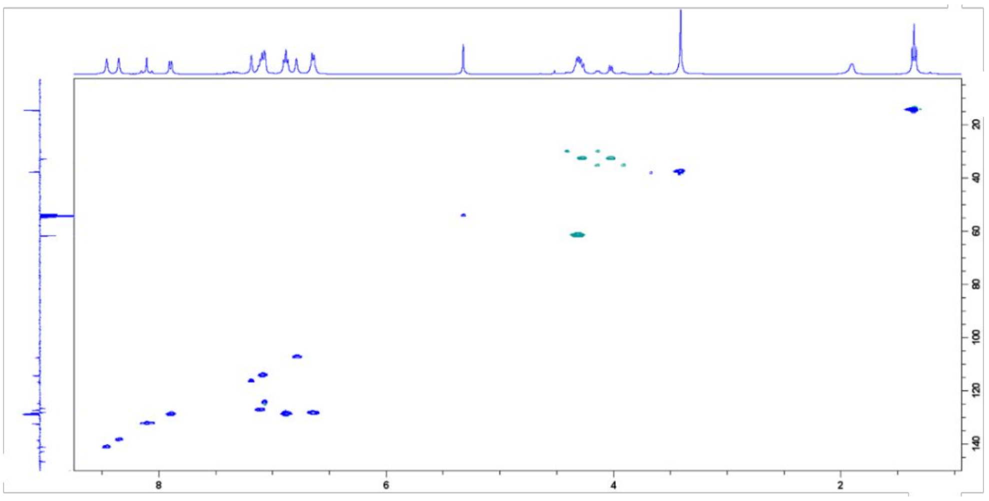

(c)

Figure S12a-c. NMR Spectra of **6a** at 248K in methylene chloride- d_2. ^{1}H (a), ^{1}H-^{1}H COSY (b), ^{1}H-^{13}C HSQC (c).

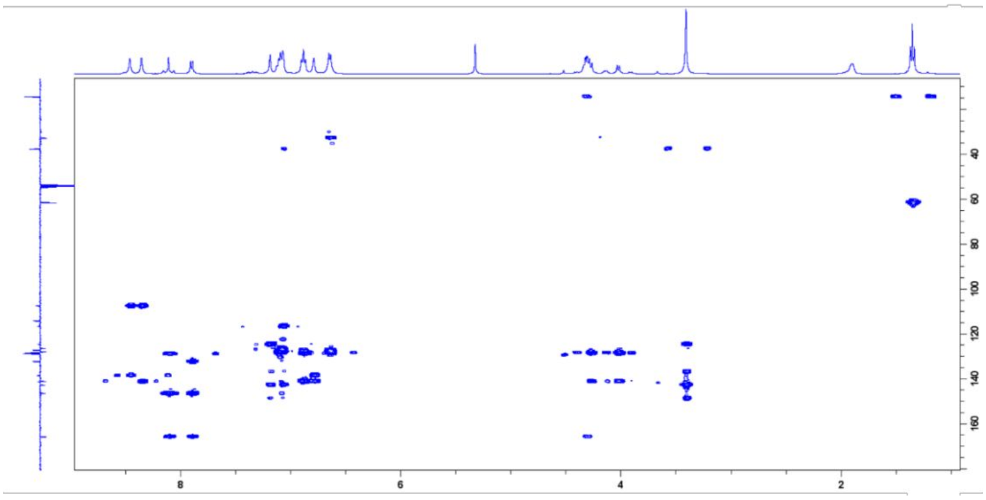

(d)

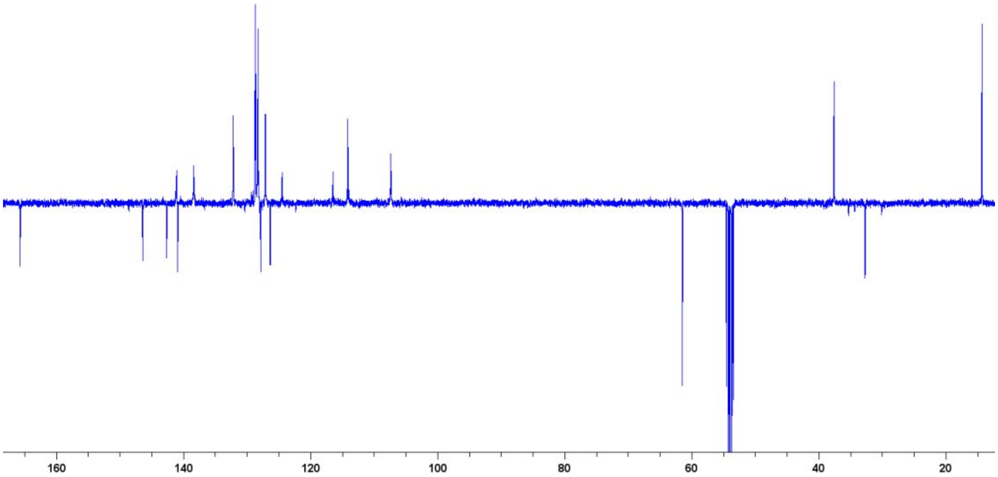

(e)

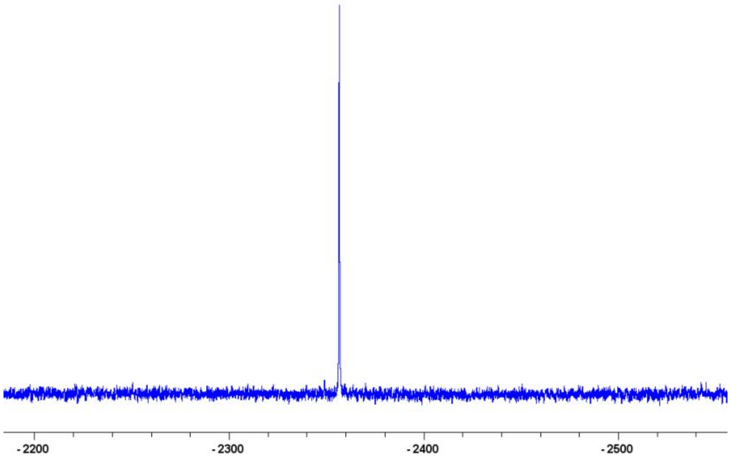

(f)

Figure S12d-f. NMR Spectra of **6a** at 248K in methylene chloride- d_2. ^{1}H-^{13}C HMBC (d), ^{13}C {^{1}H} APT (e), ^{195}Pt{^{1}H} (f).

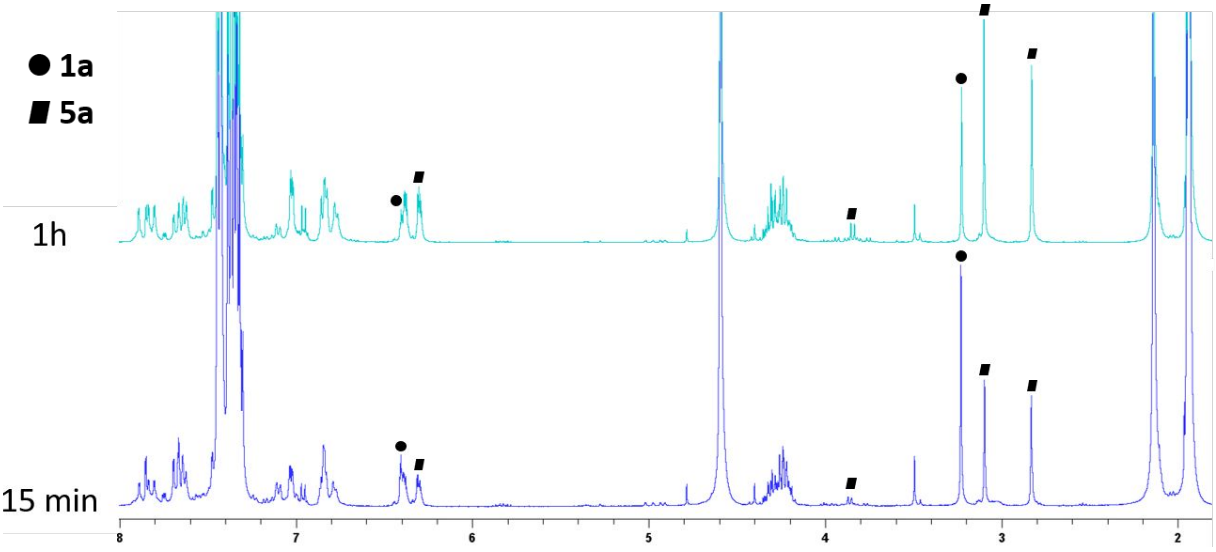

Figure S13. ^{1}H NMR spectra of the reaction of **1a + BnBr** in MeCN-d_3 in the air**.**

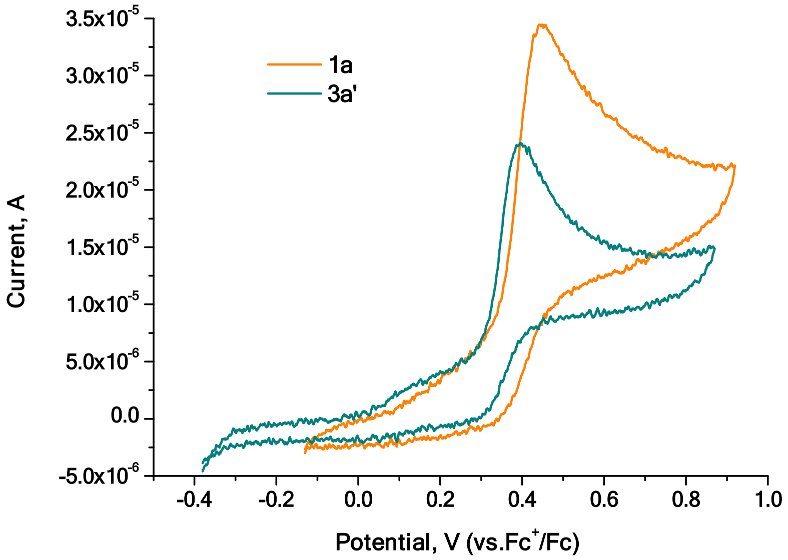

Figure S14a. Cyclic Voltammogram for oxidation of **1a** and **3a'**.

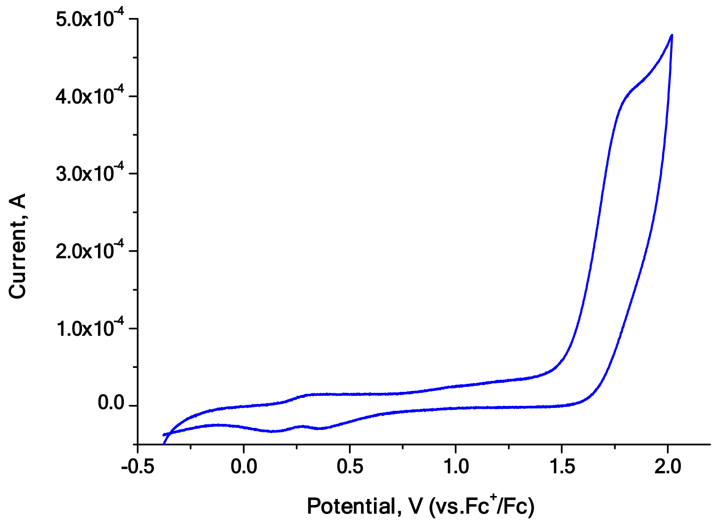

Figure S14b. Cyclic Voltammogram for oxidation of [{Pt^{III}(C^C_A*)(μ-pz)$_2$I}$_2$].

2.2. Bonding parameters of the X-ray structures

Table S2: Selected bond lengths (Å) and angles (°) for **2a** and **2b**.

	**2a**	**2b**
Pt-Pt#	3.5909(9)	3.6228(6)
Pt-C1	1.985(8)	2.003(5)
Pt-C6	2.046(8)	2.034(5)
Pt-N3	2.122(6)	2.122(4)
Pt-N41	2.077(7)	2.081(4)
Pt-Me(C17)	2.075(8)	2.081(4)
Pt-I1	2.7492(6)	2.7419(3)
C1-Pt-C6	79.9(3)	79.81(19)
C1-Pt-N3	99.3(3)	99.55(17)
C1-Pt-N41	174.8(3)	174.30(17)
C6-Pt-N3	178.0(3)	176.72(16)
C6-Pt-N41	94.9(3)	94.53(16)
N41-Pt-N3	85.9(3)	86.07(14)
C1-Pt-Me(C17)	90.3(3)	89.79(19)
C6-Pt-Me(C17)	89.8(3)	87.99(19)
C1-Pt- I1	87.7(2)	89.04(13)
C6-Pt- I1	90.3(2)	93.55(13)
Me(C17)-Pt-I1	177.9(3)	177.87(14)
Pt#1-I1-Pt	81.55(2)	82.693(13)

Symmetry transformations used to generate equivalent atoms:
#1 (**2a**) -x+1, y, -z+1/2
#1 (**2b**) -x+3/2,y,-z+3/2

Table S3: Selected bond lengths (Å) and angles (°) for **3a'** and **5a**.

	**3a'**	**5a**
Pt1-Pt2	2.6700(4)	2.6545(5)
Pt1-C1	1.997(6)	1.969(9)
Pt1-C6	2.021(6)	1.983(9)
Pt1-N5	2.072(5)	2.058(7)
Pt1-N7	2.120(5)	2.115(8)
Pt1-C33	2.067(7)	2.138(9)
Pt2-C14	1.981(6)	1.974(10)
Pt2-C19	1.998(6)	2.014(8)
Pt2-N8	2.072(5)	2.051(8)
Pt2-N6	2.093(5)	2.122(7)
Pt2-Br	----	2.6899(10)
C1-Pt1-C6	79.3(3)	79.9(4)
C1-Pt1-N5	167.7(3)	169.8(3)
C6-Pt1-N5	96.0(2)	93.3(3)
C1-Pt1-N7	99.0(2)	101.0(3)
C6-Pt1-N7	172.5(2)	170.2(3)
N5-Pt1-N7	84.2(2)	84.5(3)
C1-Pt1-C33	93.6(3)	96.0(3)
C6-Pt1-C33	88.7(3)	91.9(4)
C33-Pt1-Pt2	166.6(2)	162.0(3)
C14-Pt2-C19	80.2(3)	79.8(4)
C14-Pt2-N8	168.3(2)	168.7(3)
C19-Pt2-N8	94.8(2)	93.1(3)
C14-Pt2-N6	97.8(2)	99.8(3)
C19-Pt2-N6	173.0(2)	166.7(3)
N8-Pt2-N6	85.9(2)	85.0(3)
Pt1-Pt2-Br	-----	157.05(3)

3.- References

1. RED, C., CCD camera data reduction program *Rigaku Oxford Diffraction* **2019,** *Oxford Diffraction: Oxford, UK.*

2. Sheldrick, G. M., SHELXT - Integrated space-group and crystal-structure determination. *Acta Crystallographica a-Foundation and Advances* **2015,** *71*, 3-8.

3. Frisch, M. J.; Trucks, G. W.; Schlegel, H. B.; Scuseria, G. E.; Robb, M. A.; Cheeseman, J. R.; Scalmani, G.; Barone, V.; Petersson, G. A.; Nakatsuji, H.; Li, X.; Caricato, M.; Marenich, A. V.; Bloino, J.; Janesko, B. G.; Gomperts, R.; Mennucci, B.; Hratchian, H. P.; Ortiz, J. V.; Izmaylov, A. F.; Sonnenberg, J. L.; Williams; Ding, F.; Lipparini, F.; Egidi, F.; Goings, J.; Peng, B.; Petrone, A.; Henderson, T.; Ranasinghe, D.; Zakrzewski, V. G.; Gao, J.; Rega, N.; Zheng, G.; Liang, W.; Hada, M.; Ehara, M.; Toyota, K.; Fukuda, R.; Hasegawa, J.; Ishida, M.; Nakajima, T.; Honda, Y.; Kitao, O.; Nakai, H.; Vreven, T.; Throssell, K.; Montgomery Jr., J. A.; Peralta, J. E.; Ogliaro, F.; Bearpark, M. J.; Heyd, J. J.; Brothers, E. N.; Kudin, K. N.; Staroverov, V. N.; Keith, T. A.; Kobayashi, R.; Normand, J.; Raghavachari, K.; Rendell, A. P.; Burant, J. C.; Iyengar, S. S.; Tomasi, J.; Cossi, M.; Millam, J. M.; Klene, M.; Adamo, C.; Cammi, R.; Ochterski, J. W.; Martin, R. L.; Morokuma, K.; Farkas, O.; Foresman, J. B.; Fox, D. J. *Gaussian 16 Rev. C.01*, Wallingford, CT, 2016.

4. Zhao, Y.; Truhlar, D. G., The M06 suite of density functionals for main group thermochemistry, thermochemical kinetics, noncovalent interactions, excited states, and transition elements: two new functionals and systematic testing of four M06-class functionals and 12 other functionals. *Theor. Chem. Acc.* **2008,** *120*, 215-241.

5. Wang, Y.; Verma, P.; Jin, X.; Truhlar, D. G.; He, X., Revised M06 density functional for main-group and transition-metal chemistry. *Proc. Natl. Acad. Sci.* **2018,** *115*, 10257.

6. Grimme, S.; Antony, J.; Ehrlich, S.; Krieg, H., A consistent and accurate ab initio parametrization of density functional dispersion correction (DFT-D) for the 94 elements H-Pu. *J. Chem. Phys.* **2010,** *132*, 154104.

7. Andrae, D.; Häußermann, U.; Dolg, M.; Stoll, H.; Preuß, H., Energy-adjustedab initio pseudopotentials for the second and third row transition elements. *Theor. Chim. Acta* **1990,** *77*, 123-141.

8. Ditchfield, R.; Hehre, W. J.; Pople, J. A., Self-Consistent Molecular-Orbital Methods. IX. An Extended Gaussian-Type Basis for Molecular-Orbital Studies of Organic Molecules. *J. Chem. Phys.***1971,** *54*, 724-728.

9. Hariharan, P. C.; Pople, J. A., The influence of polarization functions on molecular orbital hydrogenation energies. *Theor. Chim. Acta* **1973,** *28*, 213-222.

10. Tomasi, J.; Mennucci, B.; Cammi, R., Quantum Mechanical Continuum Solvation Models. *Chem. Rev.* **2005,** *105*, 2999-3094.
